# A newly developed oral infection mouse model of shigellosis for immunogenicity and protective efficacy studies of a candidate vaccine

**DOI:** 10.1128/iai.00346-24

**Published:** 2024-12-18

**Authors:** Risha Haldar, Prolay Halder, Hemanta Koley, Shin-ichi Miyoshi, Santasabuj Das

**Affiliations:** 1 Division of Clinical Medicine, ICMR-National Institute of Cholera and Enteric Diseases 30170 , Kolkata, West Bengal, India; 2 Division of Bacteriology, ICMR-National Institute of Cholera and Enteric Diseases 30170 , Kolkata, West Bengal, India; 3 Division of Medicine, Dentistry and Pharmaceutical Sciences, Graduate School of Medicine, Dentistry and Pharmaceutical Sciences, Okayama University 199491 , Okayama, Okayama, Japan; 4 Division of Biological Science, ICMR-National Institute of Occupational Health 28993 , Ahmedabad, Gujarat, India; University of California Davis, Davis, California, USA

**Keywords:** oral route, *S. flexneri*, shigellosis, bivalent subunit vaccine (IpaB–T2544), mouse model

## Abstract

*Shigella* infection poses a significant public health challenge in the developing world. However, lack of a widely available mouse model that replicates human shigellosis creates a major bottleneck to better understanding of disease pathogenesis and development of newer drugs and vaccines. BALB/c mice pre-treated with streptomycin and iron (FeCl_3_) plus desferrioxamine intraperitoneally followed by oral infection with virulent *Shigella flexneri 2a* resulted in diarrhea, loss of body weight, bacterial colonization and progressive colitis characterized by disruption of epithelial lining, loss of crypt architecture with goblet cell depletion, increased polymorphonuclear infiltration into the mucosa, submucosal swelling (edema), and raised proinflammatory cytokines and chemokines in the large intestine. To evaluate the usefulness of the model for vaccine efficacy studies, mice were immunized intranasally with a recombinant protein vaccine containing *Shigella* invasion protein invasion plasmid antigen B (IpaB). Vaccinated mice conferred protection against *Shigella*, indicating that the model is suitable for testing of vaccine candidates. To protect both *Shigella* and *Salmonella*, a chimeric recombinant vaccine (rIpaB–T2544) was developed by fusing IpaB with *Salmonella* outer membrane protein T2544. Vaccinated mice developed antigen-specific serum IgG and IgA antibodies and a balanced Th1/Th2 response and were protected against oral challenge with *Shigella* (*S. flexneri 2a*, *Shigella dysenteriae*, and *Shigella sonnei*) using our present mouse model and *Salmonella* (*Salmonella* Typhi and Paratyphi) using an iron overload mouse model. We describe here the development of an oral S*higella* infection model in wild-type mouse. This model was successfully used to demonstrate the immunogenicity and protective efficacy of a candidate protein subunit vaccine against *Shigella*.

## INTRODUCTION

*Shigella* is a Gram-negative, human-restricted, bacterial pathogen that is transmitted through the feco-oral route and creates a replicative niche in the large intestine, leading to severe recto-colitis, known as shigellosis. The global incidence of the disease ranges from 80 to 165 million each year with 600,000 deaths, primarily in children under the age of 5 (1). The disease is manifested by mucoid or bloody diarrhea, fever, abdominal pain, and failure to thrive (2). Shigellosis in the endemic zones is mainly caused by *Shigella flexeneri 2a* and *Shigella sonnei*, and the colon biopsies show epithelial destruction with edema, capillary congestion, hemorrhage, crypt hyperplasia, goblet cell depletion, and mononuclear and polymorphonuclear (PMN) leukocyte cell infiltration (3). Widespread multidrug resistance results in frequent treatment failures for *Shigella* infection, while attempts to develop vaccines have yielded limited success so far.

*Shigella* is a locally invasive pathogen that replicates in the epithelial cell cytosol after exiting phagosomes and hides from the host immune system by lateral cell-to-cell spread. Bacteria reaching the lamina propria are taken up by the macrophages, wherein *Shigella* could induce pyroptosis, a mode of inflammatory cell death (2). Extensive *in vitro* and *in vivo* studies have identified that a type III secretion system (T3SS) and its multiple secreted effectors, such as invasion plasmid antigen B (IpaB), IpaC, IpgD, and IpaH7.8, play critical roles in *Shigella* pathogenesis (4). While many targets of these effector proteins have been identified in the cell culture system, their *in vivo* significance is largely unknown.

Non-availability of a suitable animal model that mimics the human disease and is genetically manipulable remains a major bottleneck in better understanding of *Shigella* pathogenesis and vaccine development. Several *in vivo* models, including guinea pig keratoconjunctivitis, rabbit ileal loop and rectal infection, newborn mouse and mouse pulmonary infection, and non-human primate and human challenge models have been reported for shigellosis (5–14). However, high cost, ethical concerns, less available immunological tools, neonate age limit, and irrelevant nature of the target organs limit their usefulness. More recently, an intraperitoneal infection model and a genetically modified (nlrc4−/−) mouse model have been established wherein the relation between *Shigella* infection and intestinal pathology was studied (15, 16). While this is a useful model to study disease pathogenesis, the genetically modified model may not be widely available, and the model’s usefulness for vaccine immunogenicity and protective efficacy was not tested.

*Shigella* colonizes the human large intestine, which is the body site for the largest number and diversity of resident flora in mammals that provides protection against pathogenic bacteria. Studies were published on Salmonella enterica serovar Typhimurium (STm) enterocolitis in mice after reduction of resident intestinal bacterial load by pre-treatment with streptomycin (17). Varying levels of intestinal inflammation were observed with several other *Salmonella enterica* serovars, but not with *Salmonella* Paratyphi after streptomycin pre-treatment (18).

Iron is an essential micronutrient for intracellular survival and growth of many Gram-negative bacteria, including *Salmonella* Typhi and *Shigella*, as it functions as a cofactor of several enzymes involved in basic biological processes such as DNA replication and respiration (19). As a part of the inflammatory response to infection, the host tends to sequester Fe from the bone marrow sites of erythropoiesis to storage, most typically to bind ferritin and induce hypoferremia, reducing the amount of free Fe available for the pathogen. The fact that an excess of free Fe in the system increases the susceptibility of the host to infection in both mice and humans highlights the protective relevance of host sequestration of Fe. The dose of Fe needed to make it susceptible to infection, however, must be given at a level high enough to inhibit neutrophil and macrophage immunological responses as well as the synthesis of tumor necrosis factor alpha (TNF-α) and nitric oxide. While this dose is toxic to the bacterium (20), Fe toxicity in mice can be eliminated with a lower dose of iron, combined with the iron chelator deferoxamine (DFO), keeping the establishment of infection intact as observed for *Salmonella* (21). DFO is a hexadentate chelator which binds with iron at a 1:1 molar ratio, thereby removing free iron from the bloodstream and enhancing its elimination in the urine (22). Bacteria can bind and take up ferrioxamine group of DFO via the FoxA receptor for utilization of iron, as was reported for *Yersinia enterocolitica* (23). FoxA is a TonB-dependent transporter of ferrioxamines (24) and TonB-dependent receptor delivers the siderophore to the periplasm where it is transported to the cytosol by the FhuBCD, a PBP-dependent ABC transporter (25). Increased iron availability has been associated with decreased abundance of more beneficial bacteria like bifidobacteria and increased infection by enteric pathogens (26). *In vitro* studies showed heightened bacterial proliferation upon transition from iron-deficient environments to those with elevated iron levels, leading to adhesion, invasion, and injury to the epithelium (27). Iron-treated chickens developed small granulomatous lesions in the lamina propria with villus epithelial shedding of the large intestine after oral challenges with *Yersinia pseudotuberculosis* (28). Furthermore, pre-treatment with iron-dextran resulted in increased susceptibility of mice to yersiniosis, when orally or parenterally challenged with *Y. enterocolitica* (29). We had previously used iron (plus iron chelator desferal) to develop a mouse model of systemic *Salmonella* Typhi infection (30).

*Shigella* IpaB is highly conserved among all *Shigella* serotypes (31). IpaB-specific antibody generates phagocytic activity (32, 33) and triggers interferon gamma (IFN-γ)-mediated immune response (34). Intranasal immunization with IpaB generated robust systemic and mucosal antibodies and T cell-mediated immunity and protected mice against lethal pulmonary infection with *Shigella flexneri* and *Shigella sonnei* (35, 36). Evidence from *Shigella* controlled human infection model (CHIM) studies showed that IpaB-specific serum IgG may also serve as a correlate of protection (37).

We report the development of an oral *Shigella* infection model in mice after combined pre-treatment with streptomycin and iron. This model was successfully used to demonstrate the immunogenicity of a subunit protein candidate vaccine against *Shigella*. We had earlier reported that subcutaneous (s.c.) immunization with a conserved *S*. Typhi/*S*. Paratyphi outer membrane protein T2544 induces protective humoral and cell-mediated immune responses in an iron overload mouse model of infection when administered through the subcutaneous route (30, 38). Furthermore, T2544 was successfully incorporated into a glycoconjugate vaccine for multivalent protection (39). Here, to provide a broader coverage against both *Salmonella* and *Shigella*, we have combined T2544 with IpaB to generate a chimeric protein (IpaB–T2544) and have shown that immunization with the recombinant chimeric protein protects mice against *Shigella* and typhoidal *Salmonella,* thus acting as a bivalent vaccine candidate.

## MATERIALS AND METHODS

### Bacterial strains, growth conditions, and plasmid

*Salmonella* Typhi Ty2 was a generous gift from J. Parkhill, Sanger Institute, Hinxton, UK. *Shigella flexneri 2a* (2457T) and clinical isolates of *Shigella dysenteriae*, *Shigella sonnei*, and *Salmonella* Paratyphi A were received from IMTECH, Chandigarh. All strains were grown in Hectoen enteric agar (BD DIFCO) and were maintained in tryptic soy agar (TSA, BD DIFCO) at 37°C. *Escherichia coli* BL21, a kind gift from Dr. Rupak K. Bhadra (CSIR-IICB, Kolkata, India), was cultured in Luria–Bertani agar at 37°C. Bacterial culture media and pET-28a plasmid were purchased from BD DIFCO and Addgene (USA), respectively. The oligonucleotides used in this study were synthesized from IDT.

### Animals

Mice were procured from the animal resources of ICMR-NICED, Kolkata, India. Animals were maintained at 25°C ± 2°C with 75% ± 2% humidity and fed sterile food and water. In this study, 6-week-old female BALB/c mice were used for the immunization. All the immunological experiments and protection study were performed at the age of 10 weeks after the completion of immunization.

### Murine oral *Shigella* infection model

For the experiments, animals (10 weeks old) were housed individually or in groups equipped with steel grid floors. Mice deprived of food and water for 4–6 h before the oral streptomycin (20 mg/mouse) treatment. Afterward, the animals were supplied with water and food *ad libitum*. Twenty hours later, the streptomycin treatment, water, and food were withdrawn again, and the mice were injected with desferrioxamine (25-mg/kg body weight) intraperitoneally. Fifteen minutes later after the desferrioxamine treatment, FeCl_3_ (0.32 mg/g of body weight) was administered via the same route before 4 h of infection (34, 35). Four hours later, the mice were treated with 5% NaHCO_3_ to neutralize the stomach acids. After 20 min of bicarbonate treatment, the mice were infected by oral gavage with *Shigella flexeneri 2a* (5 × 10^7^ CFU) resuspended in phosphate-buffered saline (PBS).

### Recovery of *Shigellae* from organs and feces

To enumerate intracellular CFU from the ceca and colons from uninfected, infected, and immunized mice, organs were isolated aseptically at different time points. Tissues were vigorously washed in PBS with gentamicin (50 µg/mL) and mechanically homogenized in 1× PBS followed by lysing with 1% Triton X-100. Lysates were serially diluted and plated on TSA plates containing 100-µg/mL streptomycin. Colonies were counted after overnight incubation at 37°C.

Fecal pellets were collected and homogenized in 2% FBS in 1 mL of PBS containing protease inhibitors. For CFU determination, serial dilutions were made in PBS and plated on TSA plates containing 100-µg/mL streptomycin.

### Histopathology

Colon and cecum tissues were excised from mice and preserved in 10% formalin. Formalin-dissolved tissues were then embedded in paraffin blocks. Tissues fixed in paraffin were divided into 5-μm-thick sections. The sections were soaked in xylene twice for 20 min at 56°C to deparaffinize and were dehydrated with ethanol (twice at 100%, once at 95%, and once at 75% ethanol) for 5 min. Tissue sections were mounted on glass slides and stained with hematoxylin and eosin. Slides were examined under a microscope and evaluated in a blinded fashion by two independent investigators. Different magnifications of images were captured to represent different parameters: ×10, presence of swelling (edema) in the submucosal layer; ×20, degenerative changes in the epithelial lining, ×40, presence of increased infiltration of lymphocytes in the mucosa and submucosa, and loss of crypt architecture with loss of goblet cells. Slides were scored for different histological parameters as described in Tables S1 and S2.

### Cytokine estimation from intestinal tissue

Cecum and colon were isolated from mice at different post-infection (p.i.) time points and were rinsed five times in PBS to remove fecal contents. Organs were then homogenized in 1 mL of 2% FBS in PBS with protease inhibitors and spun down at 2,000 × *g*. The concentrations of interleukin (IL)-1β, TNF-α, IFN-γ, and CXCL10 were measured in the supernatant using the commercial enzyme-linked immunosorbent assay (ELISA) kits (R&D) following the manufacturer’s protocol. Briefly, 96-well plates were incubated overnight with capture antibody in coating buffer. Following three subsequent washes, the plates were subjected to blocking for 1 h at room temperature. Samples were added to the wells following the wash and incubated for 2 h at room temperature. After three subsequent washes, the plates were further incubated for 2 h at room temperature with detection antibody. After three washes, the plates were further incubated with streptavidin–horseradish peroxidase (HRP) for 20 min at room temperature. Following three washes, the plates were finally incubated with substrate for 30 min at room temperature. The reaction ended with the stop solution, and the color development was evaluated using spectrophotometry at 450 nm.

### Cloning expression and purification of rIpaB–T2544

*ipab* and *t2544* genes were cloned in tandem into the prokaryotic expression vector pET28a. We first amplified the 801-bp domain regions of the *ipab* gene by PCR, using the genomic DNAs of *Shigella flexeneri 2a* strain as the template, and cloned it into the pET28a vector using the following IpaB primers: IpaB forward primer, CGCGGATCCGGTGGCGGTGGCTCG GATCTTACTGCTAACCAAAAT, and IpaB reverse primer, CCGGAGCTC AACACAACCCATTACTCTGTTGAG, where BamHI and SacI were used as restriction enzymes (New England Biolabs). Later on, *t2544*, along with an upstream linker sequence (GGACCAGGACCA is the gene codon of a non-furin linker containing glycine and proline amino acids) (total sequence length 663 bp), was PCR amplified, and the PCR product was cloned between SalI and XhoI restriction enzymes (New England Biolabs) of the pET28a–IpaB plasmid (T2544 FP: GCCGTCGACGGACCAGGACCAGAAGGGATCTATATCACCGGG and T2544 RP: GCCCTCGAGTTAAAAGGCGTAAGTAATGCCGAG). After clone confirmation by restriction digestion followed by sequencing (Agri genome), the recombinant plasmid (rIpaB–T2544) was transformed into *E. coli* BL21 (DE3) pLysS cells. Transformed *E. coli* BL21 pLysS cells were inoculated into terrific broth (BD DIFCO) and incubated until the OD_600_ reached 0.5. Transformed cells were induced with 1-mM isopropylthiogalactoside for 4 h at 37°C, followed by centrifugation at 5,000 × *g*. The resulting pellet was resuspended in lysis buffer containing 8-M urea, 200-mM NaCl, 2-mM imidazole, and 50-mM Tris–HCl. Induced bacterial cells were subjected to 5–80 cycles of sonication on ice, with each cycle consisting of five pulses of 1 s each and a 1-min incubation period. The power output was designed to deliver a maximum of 30 W at a frequency of 20 kHz. The sonicated sample was purified by affinity chromatography using Ni-NTA slurry according to the manufacturer’s instructions (Qiagen). Briefly, the sonicated sample was centrifuged at 15,000 rpm for 30 min at 4°C. The supernatant was collected and used for protein purification. One milliliter of Ni-NTA slurry was centrifuged at 1,500 × *g*, and the supernatant were removed. The Ni-NTA beads were then mixed with equilibration buffer (20-mM Tris–HCl, 400-mM NaCl, 5-mM imidazole, and 8-M urea) for 1 h at room temperature. Four milliliters of protein supernatant was added to the equilibrated Ni-NTA beads and incubated at room temperature for 4 h. The protein–Ni-NTA mixture was loaded into the column (Bio-Rad empty polypropylene column), and column-bound recombinant protein was eluted using elution buffer (20-mM Tris–HCl, 300-mM NaCl, 500-mM imidazole, and 8-M urea). After that, the protein solution was renatured by gradual removal of urea by dialysis in 20-mM Tris–HCl, 150-mM NaCl, 10% glycerol, 0.5% Triton X-100, and 2-M urea buffer using a 10-kD membrane (Millipore). The purity of the protein was determined by sodium dodecyl sulfate–polyacrylamide gel electrophoresis (SDS–PAGE).

### Circular dichroism

One milliliter of rIpaB, rT2544, and rIpaB–T2544 at a concentration of 180 µg/mL was filtered through a 0.45-µm-pore size filter to remove suspended particles, if any, and was taken in a 0.1-mm-path length quartz cuvette. Circular dichroism (CD) spectrum of the protein sample was captured at the wavelength range of 200–300 nm at 25°C on the Jasco-1500 spectrophotometer. A minimum of three spectra were recorded in 1-nm steps at a speed of 50 nm/min. Baselines were subtracted, and data were recorded as ellipticity (CD [mdeg]).

### Western blot

In 12% SDS-PAGE, rT2544 (5 µg), rIpaB (7 µg), and rIpaB–T2544 (3 µg each) were resolved and then transferred to a polyvinylidene fluoride membrane (Millipore). After transfer, the membrane was washed with 1× TBS for 5 min at room temperature. After blocking with 5% bovine serum albumin (BSA) for 1 h at room temperature, membranes were washed with 1× Tris-buffered saline, pH 7.5, containing 0.1% Tween 20 (TBS-T) (vol/vol) three times for 5 min at room temperature. The membrane was then incubated with mouse anti His-tag (CST) antibody (1:1,000) in 5% BSA, 1× TBS, and 0.1% Tween 20 at 4°C with gentle shaking overnight. Membranes were washed with TBS-T five times and incubated with rabbit anti-mouse IgG antibody (1:10,000 dilutions) conjugated to HRP for 1 h at room temperature. After three washes with TBS-T in an orbital shaker, membranes were developed by chemiluminescent reagents (SuperSignal West Pico, Thermo Fisher), and the signals were captured in ChemiDoc MP imaging system (Bio-Rad).

### Animal immunization and infection

Female BALB/c mice (6 weeks old) were immunized intranasally with PBS (vehicle), 40 µg of rT2544, rIpaB, or rIpaB–T2544 at 2-week intervals three times. On days 0, 14, 28, 38, and 90 after vaccination, blood samples were collected from the mice by tail snip. The samples were then incubated for 30 min at 37°C, centrifuged for 15 min at 1,200 × *g* at 4°C, and stored at −80°C. Fecal samples were collected on days 0, 14, 28, and 38, weighed, and thoroughly dissolved in 100 mg/mL of PBS with 1% BSA (SRL). Samples were then centrifuged at 15,000 × *g* for 10 min at 4°C, and protease inhibitor cocktails (Sigma-Aldrich) were added to the supernatants before being stored at −80°C. Intestinal washes were collected after sacrifice of the mice (on day 38). To this end, the small intestine was removed and washed three times with 1 mL of PBS–1% BSA (BSA, SRL). After centrifuging the samples for 10 min at 10,000 × *g* at 4°C, protease inhibitor cocktails (Sigma-Aldrich) were added to the supernatants and stored at −80°C. Fourteen days after the last immunization, different groups of mice were infected with different bacterial strains (*Shigella* spp., *Salmonella* Typhi, and *Salmonella* Paratyphi A).

For *Shigella* infection, 14 days after the last immunization (day 42), mice were pre-treated with streptomycin sulfate and iron with desferal (desferrioxamine) (as described above) before the oral dosage of different *Shigella* strains (*Shigella flexeneri 2a* [5 × 10^9^ CFU], *Shigella dysenteriae* [5 × 10^8^ CFU], and *Shigella sonnei* [5 × 10^8^ CFU]). Infected mice were monitored for 20 days.

For *Salmonella* infection, on day 42 of first immunization, BALB/c mice were deprived of food and water for 10 h. Mice were treated with intraperitoneal injection of Fe^3+^ as FeCl_3_ (0.32 mg/g of body weight), along with desferrioxamine (25 mg/kg of body weight), 4 h prior to the bacterial challenge. Four hours later, mice were treated 5% NaHCO_3_ to neutralize the stomach acids. After 20 min of the bicarbonate treatment, mice were orally infected with 5 × 10^7^ CFU (10× LD_50_) of *S*. Typhi or 5 × 10^5^ CFU (10× LD_50_) of *S*. Paratyphi A and monitored for 10 days (34, 35).

### Cytokine estimation from splenocytes

PBS- and rIpaB–T2544-immunized mice were sacrificed on day 38, 10 days after the last immunization. Splenocytes were collected from the spleen, and 200 µL of splenocytes was cultured in RPMI 1640 medium (1 × 10^5^ cells/well) in 96-well microtiter plates. Cells were incubated with 5 µg/mL of rIpaB–T2544 for 48 h. Levels of cytokine secretion were measured (pg/mL) from the supernatants at 48 h. Cytokines were estimated by commercial ELISA kits (BD Bioscience [IL-4, IL-5, IL-6, and IL-12] and R&D [IL-4, IFN-γ, and TNF-α]) following the manufacturer’s instructions.

### ELISA

Microtiter plates were coated with 2 µg/mL of rT2544, IpaB, or rIpaB–T2544 and incubated at 4°C overnight. After rinsing with PBS containing 0.05% Tween 20, the wells were blocked using PBS containing 1% BSA (SRL) for 1 h at room temperature. Following further washes, plates were incubated with serum, feces, or intestinal lavage samples, diluted serially (1:200 to 1:409,600 for IgG and 1:20 to 1:25,600 for IgA) for 2 h at room temperature. Subsequently, rabbit anti-mouse IgG (1:10,000) or goat anti-mouse IgA (1:5,000) antibodies conjugated to HRP were used to incubate the wells for 1 h at room temperature. The immune complex was developed using tetramethyl benzidine substrate (BD OptEIA), and OD_450_ of the mixture was measured in a spectrophotometer (Shimadzu, Japan).

Details of the reagents are listed in Table S3.

### Statistical analysis

Data related to CD were analyzed using ORIGIN software (2019b). Antibody titers were represented as reciprocal of the log 2 values. GraphPad Prism version 8.0.1.244 was used for statistical analyses, and for the comparison of the two groups, Student’s *t*-test was used (*P, 0.05, **P < 0.01, ***P < 0.001, ****P < 0.0001). One-way analysis of variance (ANOVA) and two-way ANOVA with Tukey’s post-test were performed for multiple comparisons (*P < 0.05, **P < 0.01, ***P < 0.001).

## RESULTS

### Combined treatment with streptomycin and iron (FeCl_3_) renders mice susceptible to oral *Shigella* infection and disease development

**Fig 1:**
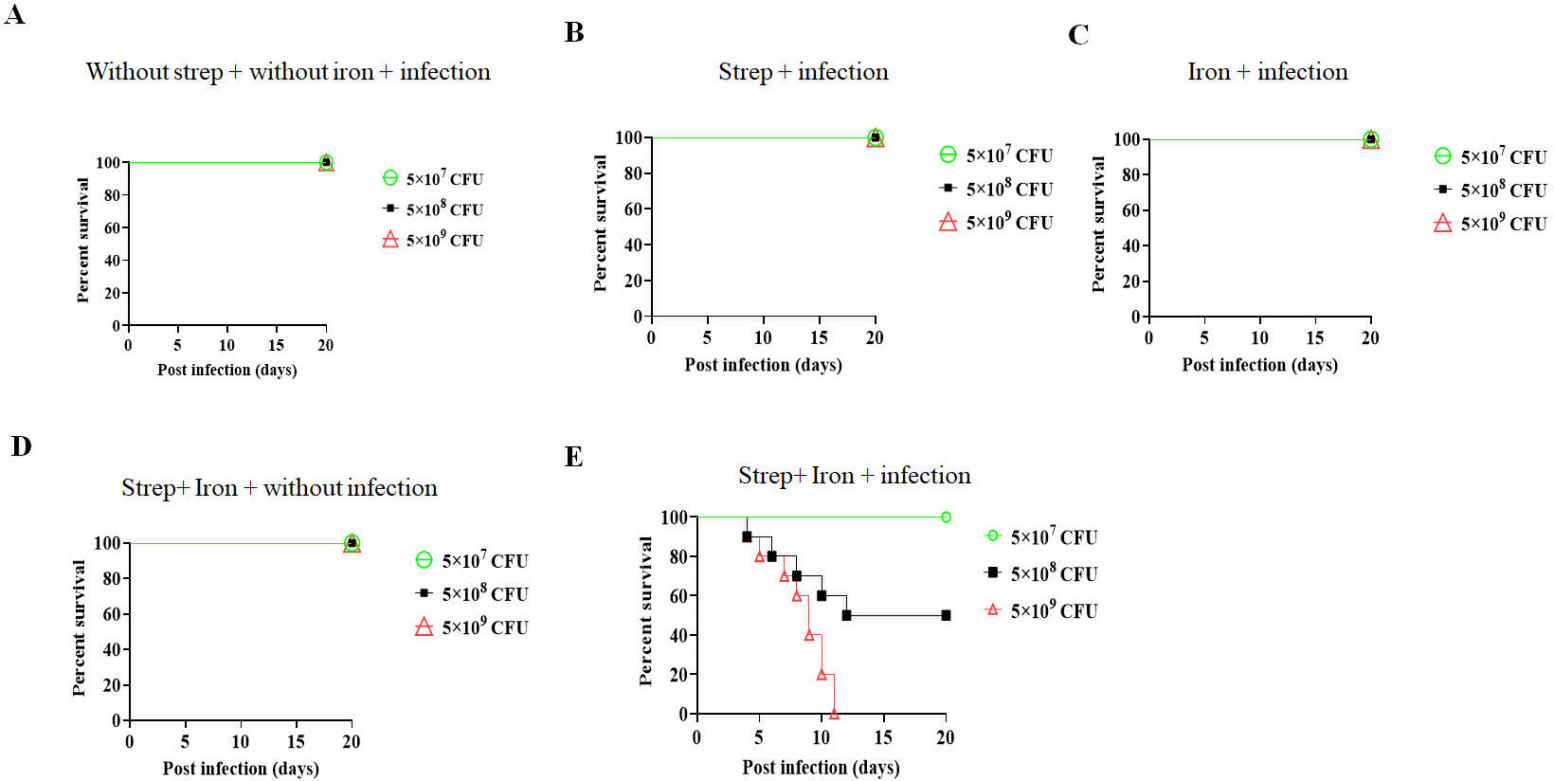
Survival assay with different doses of *Shigella flexeneri 2a*. Kaplan–Meyer plot of cumulative mortality of BALB/c mice (*n* = 6). Different groups of mice were infected (**A–C, E**) with *Shigella flexeneri 2a* with different doses, while one group remained uninfected (**D**). The mortality of mice was observed for 20 days. The color scheme used to mark different bacteria doses is as follows: green, 5 ×10^7^ CFU; black, 5 × 10^8^ CFU; and red, 5 × 10^9^ CFU.

To establish an oral *Shigella* infection model, different groups of BALB/c mice (10 weeks old, *n* = 6) were pre-treated with various combinations of oral streptomycin and/or injectable iron (FeCl_3_) plus iron chelator, desferal (FeCl_3_/desferal), 24 and 4 h, respectively, before the oral gavage with different doses of virulent *Shigella flexeneri 2a* as shown in Fig. 1. All mice that received either streptomycin or FeCl_3_/desferal survived the bacterial challenge (Fig. 1A through C). In contrast, 50% and 100% of the mice that received 5 × 10^8^ CFU (LD_50_) and 5 × 10^9^ CFU (LD_100_), respectively, after pre-treatment with both streptomycin and FeCl_3_/desferal succumbed to the infection. All the mice similarly pre-treated, but challenged with 5 × 10^7^ CFU (sublethal dose) of *S. flexneri 2a* survived (Fig. 1). To investigate the difference in the outcome of infection with the lower (5 × 10^7^ CFU) and the higher (5 × 10^8^ CFU) doses of *S. flexneri 2a*, we measured the body weights daily and fecal shedding of bacteria on alternate days of the infected mice until 14 days post-infection (Fig. S1A and B). The results showed a sharp decline in the body weights of all the infected mice in the first 2 days after the infection. However, mice infected with the lower dose rapidly gained weight after this time point, while those infected with the higher dose displayed a much slower gain of weight, suggesting greater impact of infection. Comparison of fecal shedding between the two groups of mice showed maximum shedding after 2 days of infection, which was similar for both groups. This suggests greater (~10-fold) colonization of the intestine by *S. flexneri* for the mice that received the higher dose. This corroborated with more rapid decline of bacterial shedding in the animals infected with the lower bacterial dose to touch the baseline within 10 days. In contrast, mice infected with the higher dose showed a much slower decline of fecal bacterial shedding. Thus, the difference in deaths of mice between the two groups may be explained by significantly better and longer infection of the mice that received 10 times more bacteria. The above results underscored that although there was no bottleneck for infection, a threshold of infectious dose was required to cause severe diseases, resulting in death. Given that the higher dose (5 × 10^8^ CFU) used here was the LD_50_ dose, only 50% of the mice died, and this occurred over a period of time. This difference was most likely related to the success of infection that varies for experiments with live organisms, especially mammals. This is suggested by the fact that bacterial shedding of the surviving mice from this group gradually decreased over time, indicating that mice which were colonized by a fewer number of bacteria or handled the infection better survived longer. Overall, these findings suggested that iron plus streptomycin pre-treatment increases susceptibility of mice to oral *Shigella* infection. Furthermore, LD_50_ and LD_100_ doses for other *Shigella* serovars, such as *S. dysenteriae* and *S. sonnei*, in streptomycin plus FeCl_3_/desferal pre-treated mice were 1 log lower than *S. flexneri 2a* (Fig. S2).

**Fig 2:**
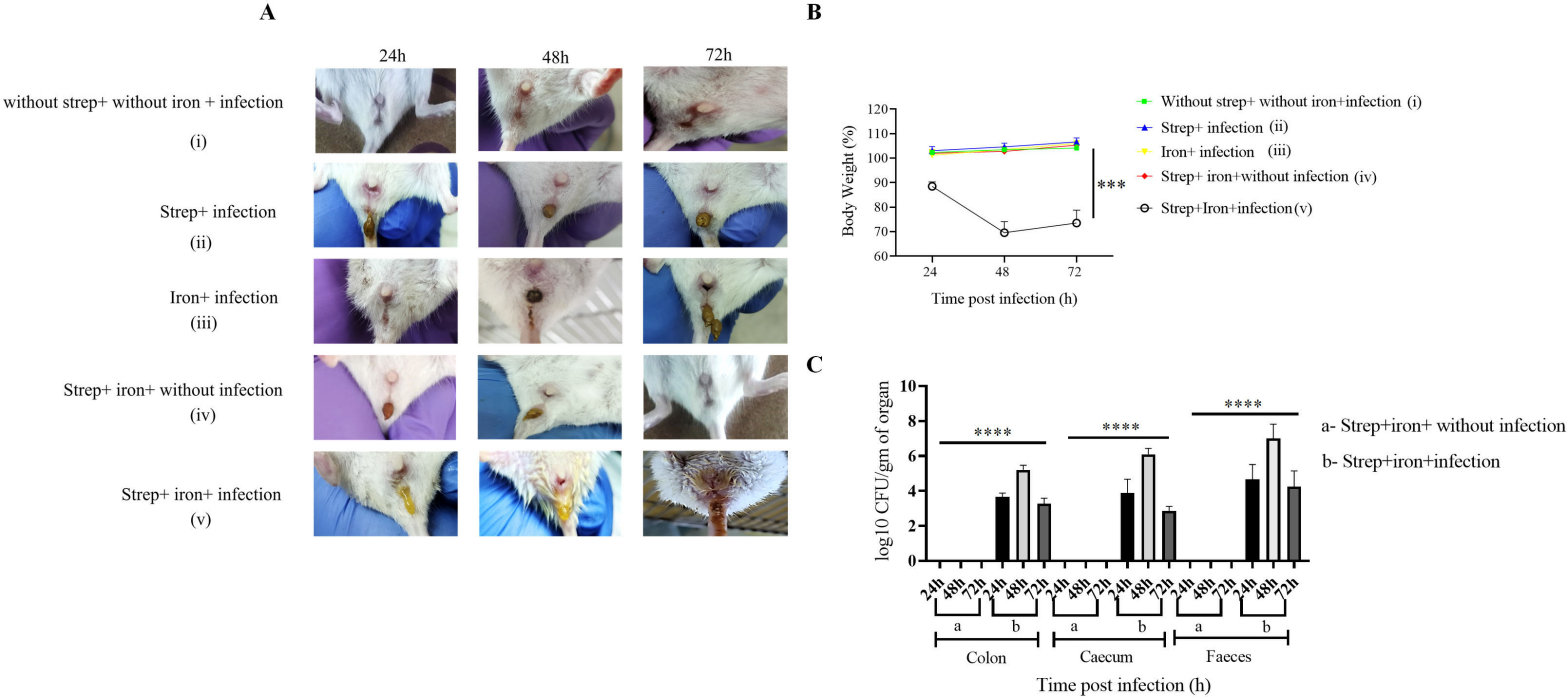
Adult mice were susceptible to oral infection with *S. flexneri 2a* strain at a dose of 5 × 10^7^. BALB/c mice were orally infected with 5 × 10^7^ dose of *S. flexneri 2a*. (**A**) Photographs of the anal region of BALB/c mice of different groups at different post-infection time points. A representative image from three independent experiments (*n* = 4 mice per group) is shown. (**B**) Body weight changes post-infection. Data represent mean ± SEM values of multiple animals (*n* = 4 mice per group) at each time point. Statistical analyses were performed by one-way ANOVA and Tukey’s post-test for multiple comparisons. ***P < 0.001. (**C**) Colony forming units (CFU) of *S. flexneri 2a* in the colon, cecum, and feces of infected and control mice at different post-infection time points. All the tissue homogenates were cultured overnight on TSA plates. Data represent mean ± SEM of the values from multiple animals (*n* = 6 mice per group). Statistical analyses were performed by one-way ANOVA and Tukey’s post-test for multiple comparisons. ****P < 0.0001.

Furthermore, sublethal infection (5 × 10^7^ CFU) with *S. flexneri 2a* led to diarrhea by 24 h (Fig. 2A, panel v) and maximum body weight loss (69%) at 48 h p.i. (Fig. 2B, panel v) in the streptomycin and iron pre-treated group. In comparison, mice from the comparator groups, which either received no infection or combined treatment with FeCl_3_ (plus desferal) and streptomycin had no diarrhea (Fig. 2A, panels i through iv) and showed continued gain in body weights (Fig. 2B, panels i through iv).

In the infected mice, disease severity correlated with bacterial recovery from the colon and cecum lysates and the feces. We observe peak CFU at 48 h p.i. that gradually decreased with time (Fig. 2C), indicating gradual recovery, which corroborated with the self-limiting *Shigella* gastroenteritis of humans (40). In our study, we have used only virulent strains shed in the stool that were Congo red positive, suggesting that they contain virulence plasmid. Together these results suggested that oral challenge with *Shigella* spp. readily causes colonic infection and disease in streptomycin- and iron-pre-treated mice and may be considered as a model for human shigellosis.

### Oral *S. flexneri 2a* infection of mice pre-treated with streptomycin and iron causes colitis

**Fig 3:**
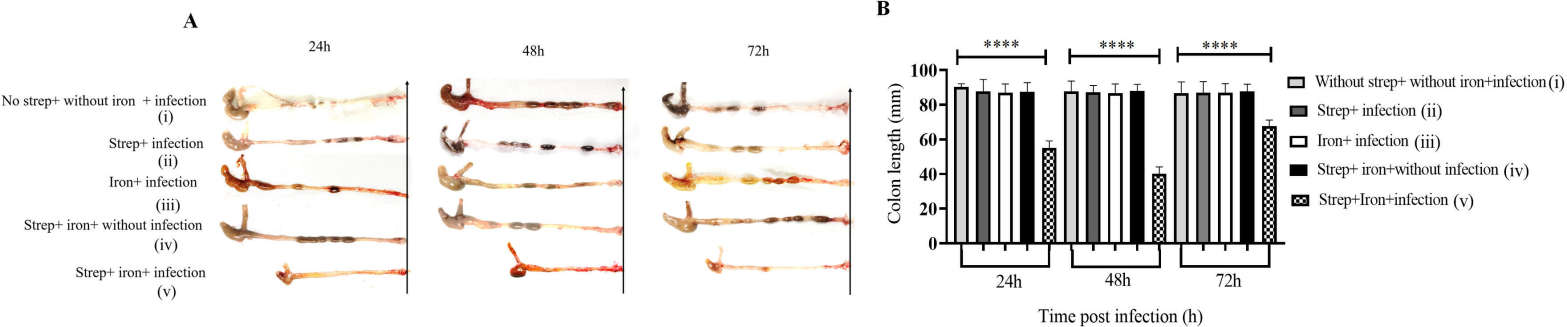
Susceptible adult mice showed decreased colon length after oral infection with *S. flexneri 2a* strain at a dose of 5 × 10^7^. (**A**) Colonic shortening after the infection. Cecum and ascending colon isolated from the infected and uninfected mice, sacrificed at different time points post-infection. A representative image from three independent experiments (*n* = 6 mice per group) is shown. (**B**) Graphical representation of colon length. Data represent mean ± SEM of the values from multiple animals (*n* = 6 mice per group). Statistical analyses were performed using one-way ANOVA and Tukey’s post-test for multiple comparisons. ****P < 0.0001.

Length of the colon is a widely used indicator of the severity of *Shigella* colitis. We observed maximum shortening of the colon (average length 40 mm) at 48 h post-infection only in strep + iron pre-treated animals (Fig. 3A, panel v and B) in comparison to >87 mm in the other groups (Fig. 3A and B i-iv).

**Fig 4:**
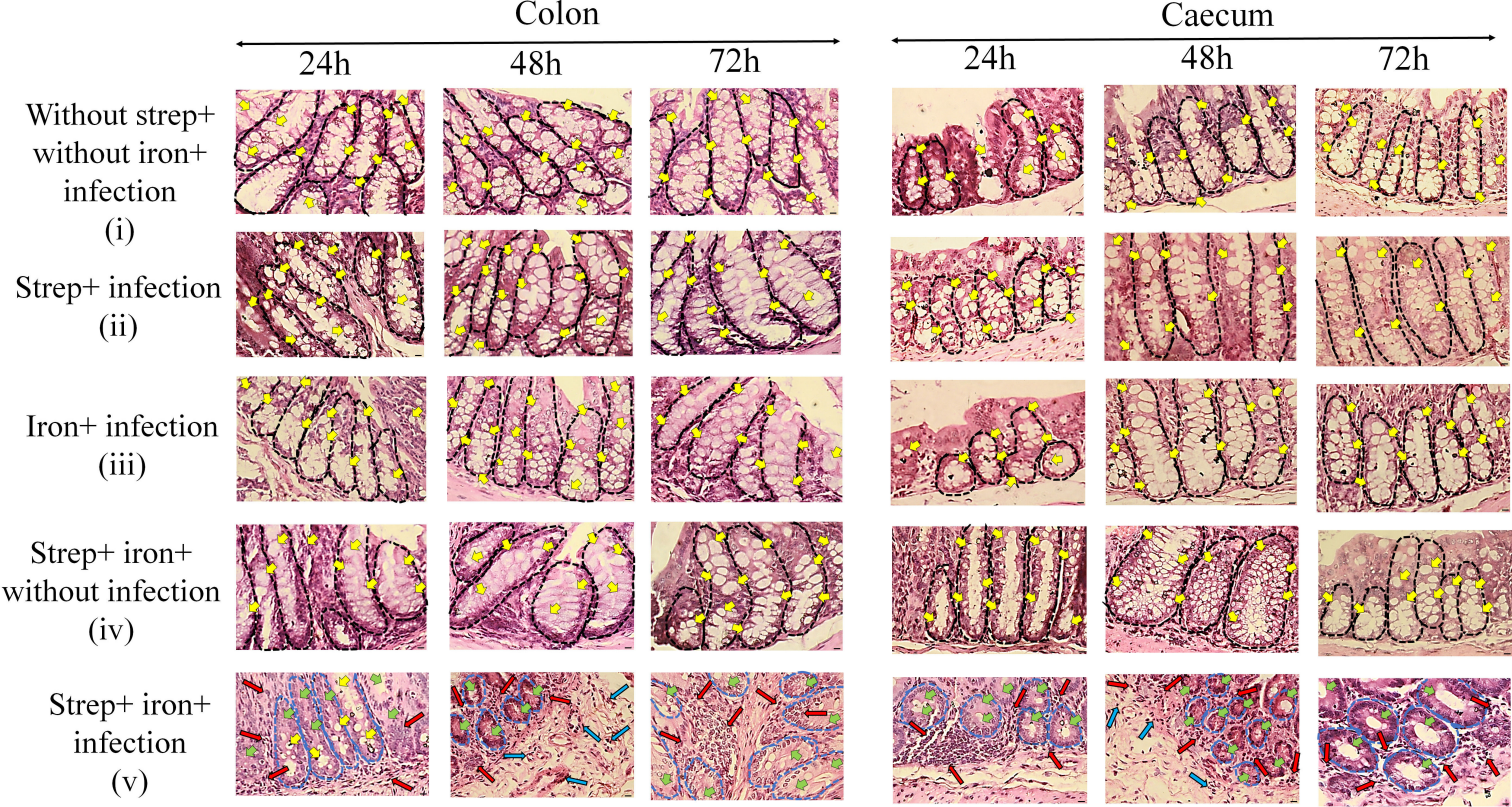
Histological sections of the colon and cecum tissue of BALB/c mice after different treatments. Different groups of mice (*n* = 6 mice per group) were infected with 5 × 10^7^ CFU *Shigella flexeneri 2a* and sacrificed at the indicated time points. Colon and cecum were excised, fixed, and embedded in paraffin. Tissue sections were stained with hematoxylin and eosin and observed under a microscope (×40 magnification, scale bar = 10 µm). The images are the magnified form of the ×10 images (areas of green box) presented in the supplemental information. Intact crypt architecture with abundant records of goblet cells and intact mucosa and submucosa without abnormal infiltrates was observed in the control group of mice (i–iv). Streptomycin–iron with infected group (v) showed loss of crypt architecture with decreased goblet cells and increased infiltration of lymphocytes in the mucosa and submucosa. Black round dotted circles represent intact crypt architecture, and blue round dotted circles represent loss of crypt architecture. Different colors of arrows indicate different parameters as follows: yellow, abundant goblet cells; green, loss of goblet cells; red, lymphocyte infiltration in the mucosa; blue, lymphocyte infiltration in the submucosa.

Colon and cecum morphology were studied by histology of hematoxylin and eosin-stained tissues after *Shigella flexeneri 2a* infection (5 × 10^7^ CFU) (Fig. 4). Tissue sections of the control group (Fig. 4, panels i through iv; Fig. S3, S4 [panels i through iv], and S5 [panels i through iv]) showed well-organized histological features with intact intestinal epithelial lining and properly arranged crypts with abundant goblet cells. No abnormal PMN infiltrates and thickening of the mucosal and submucosal layers were observed. In contrast, streptomycin and iron/desferal pre-treated infected mice showed evidence of tissue damage, characterized by disruption of epithelial lining, loss of crypt architecture with decreased goblet cell numbers, and increased PMN infiltration into the mucosa of the colon and cecum at 24 and 72 h (Fig. 4 [panel v]; Fig. S4 [panel v] and S5 [panel v]). A submucosal swelling (edema) with PMN infiltration was observed at 48 h of infection. Resolution of these damages was visible after 72 h with recovery of crypt structure and goblet cell number, decreased PMN infiltration, and repair of the epithelial lining. The process of resolution and repair further progressed, and the recovery of the tissue architecture was complete by 7 days post-infection (Fig. S6 and S7). A blind histological scoring was performed with the infected tissue samples and plotted in a bar diagram (Tables S1 and S2; Fig. S8).

In our study, visible bacteria within the cells were observed in the pathological specimens (Fig. S6 and S7; 24–72 h, white arrows, ×40 magnification).

**Fig 5:**
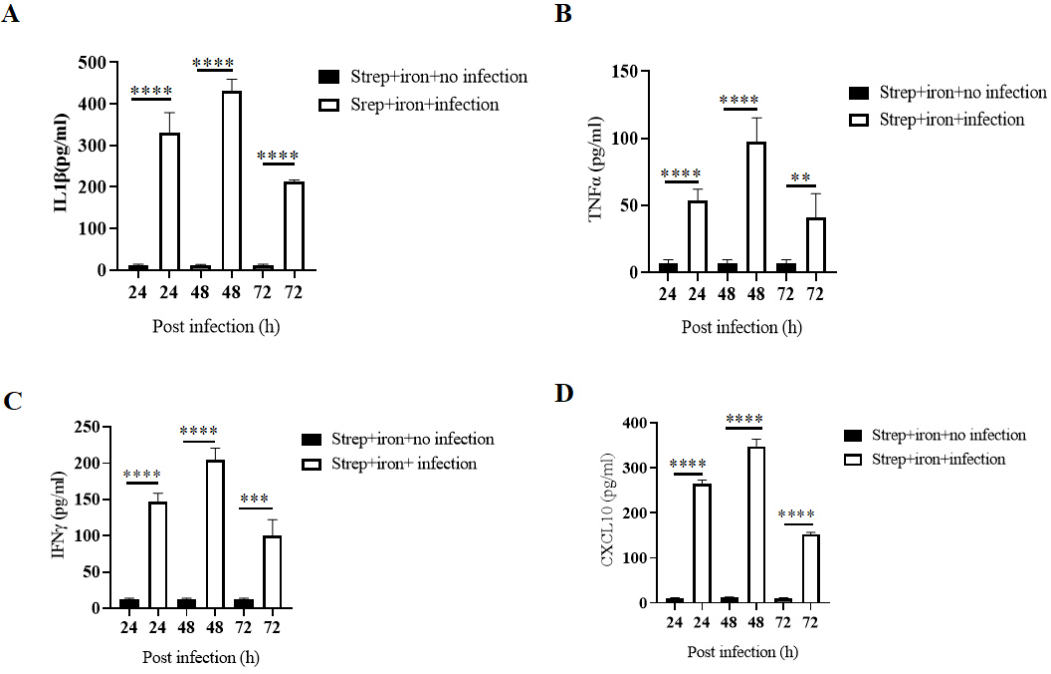
Proinflammatory cytokine and chemokine induction after *Shigella flexeneri 2a* infection. Streptomycin and iron pre-treated BALB/c mice (*n* = 4) were infected with 5 × 10^7^ CFU *Shigella flexeneri 2a*. ELISA performed with the colonic tissue homogenates for the proinflammatory cytokines (**A–C**) and chemokines (**D**). Data represent mean ± SEM values from different mice samples (*n* = 4). Statistical analyses were performed with two-tailed Student’s *t*-test between two different groups. **P < 0.01, ***P < 0.001, ****P < 0.0001.

Together these data suggested that oral *Shigella* infection caused severe inflammatory damage to the colonic tissues in streptomycin- and iron-pre-treated mice. The macroscopic and microscopic features of colonic and cecal inflammation were accompanied by the release of high levels of proinflammatory cytokines and chemokines, such as TNF-α, IL-1β, IFN-γ, and CXCL10 in the supernatants of the tissue homogenates at different time points with maximum levels recorded after 48 h of infection (Fig. 5A through D). Together the above results suggested that streptomycin and iron pre-treatment of adult mice rendered them susceptible to oral *Shigella* infection, with rapid colonization of the large intestine and the development of progressive colitis, manifested by diarrhea, loss of body weight, shortening of the colon with tissue destruction, and proinflammatory cytokine and chemokine production.

### Intranasal immunization with recombinant IpaB augmented humoral immune response and protected mice against oral *Shigella* infections

**Fig 6:**
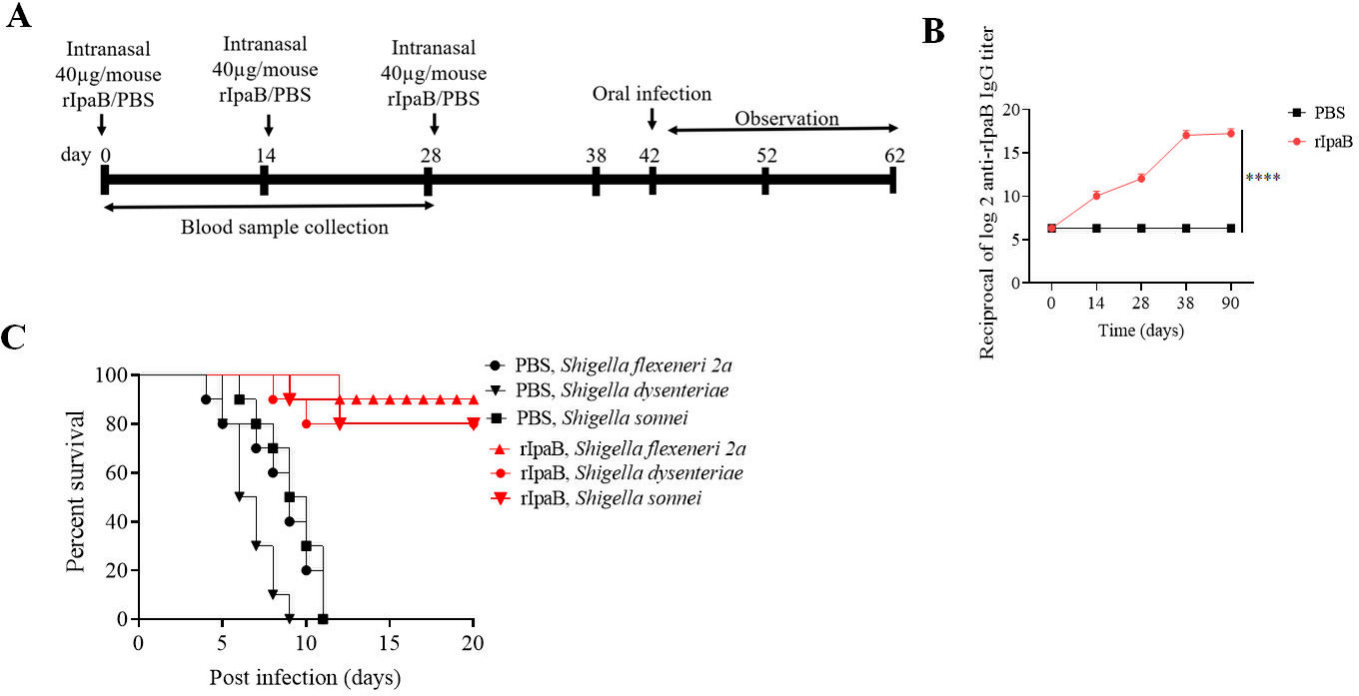
Intranasal immunization with recombinant IpaB augmented humoral immune response and protected mice against oral *Shigella* infections. (**A**) Experimental schedule of immunization, sample collection, and bacterial challenge of BALB/c mice. (**B**) Time kinetics of antigen-specific total IgG in the mouse serum as measured by ELISA. Data represent mean ± SEM values from different mice samples (*n* = 5). *X*-axis indicate the time points after the start of the immunization when samples were collected. Statistical analysis was performed using two-way ANOVA and Tukey’s post-test for multiple comparisons. ****P < 0.0001. The color scheme used to mark different experimental groups are as follows: black PBS; red, rIpaB. (**C**) Kaplan–Meyer plot of cumulative mortality of the mice immunized intranasally with vehicle or the recombinant protein. Ten days after the last immunization (38 days), immunized mice were pre-treated with streptomycin and iron followed by oral infection with different *Shigella* spp. (*Shigella flexeneri 2a* [5 × 10^9^ CFU, *n* = 10], *Shigella dysenteriae* [5 × 10^8^ CFU, *n* = 10], and *Shigella sonnei* [5 × 10^8^ CFU, *n* = 10]) and monitored for 20 days. The color scheme used to mark different experimental groups are as follows: black, PBS; red, rIpaB.

To determine if the new oral shigellosis model was suitable for vaccine efficacy studies, we generated a recombinant protein (rIpaB)-based subunit vaccine by expressing a gene construct of *Shigella flexneri 2a ipab* (801 bp) in *E. coli*. The recombinant protein (rIpaB, 37 kDa) was purified from *E. coli* BL21 (DE3) PlyS by affinity chromatography using Ni-NTA agarose (Fig. S9C), and its purity and size were confirmed by Western blots (Fig. S9D). Secondary structure of rIpaB–T2544 was analyzed by far-UV CD spectra that showed one negative band, indicating alpha helix, and one positive band, suggesting β helical structure at 222.6 and 202.3 nm, respectively (Fig. S9E). To check for the protective efficacy of the candidate vaccine, BALB/c mice were immunized intranasally with three doses of the recombinant protein or the vehicle (PBS) at 14-day intervals (Table S4; Fig. 6A). Analysis of serum antibody endpoint titers showed that immunization with rIpaB induced 2,560 times higher IpaB-specific IgG compared with the vehicle (PBS) immunization (Fig. 6B). Oral infection with 10× LD_50_ dose of the *Shigella* spp. killed all the mice that received the vehicle within 11 days of infection, whereas 80%–90% of the mice immunized with rIpaB were still alive beyond 20 days (Fig. 6C). Collectively, the above data indicated that the newly developed oral *Shigella* infection model could be effectively used to evaluate the protective efficacy of vaccine candidates.

### Recombinant IpaB immunization reduces colonization of the mouse large intestine by S*higella flexneri 2a* and disease manifestations after oral infection

**Fig 7:**
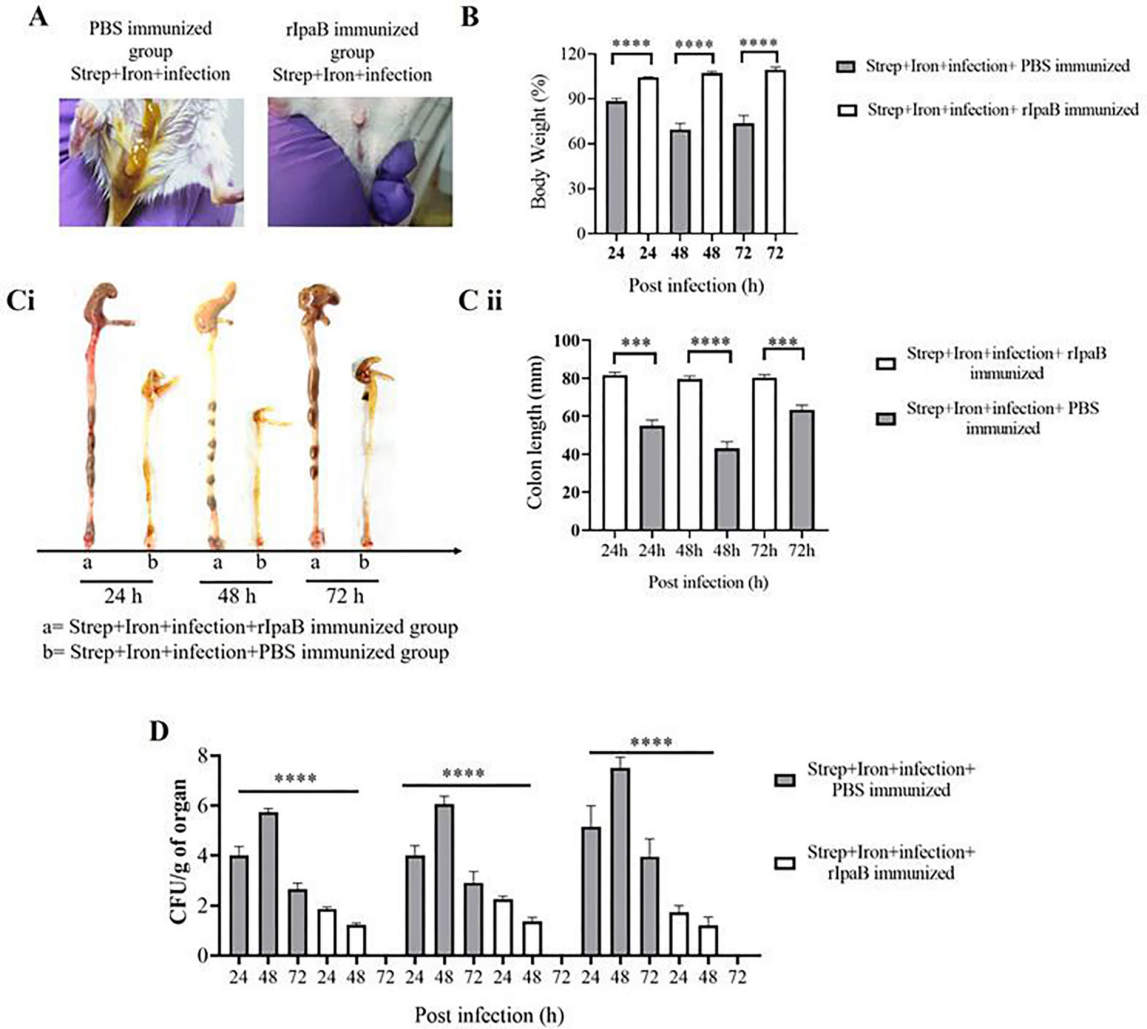
rIpaB-immunized mice were not susceptible to infection following oral administration of *S. flexneri 2a*. BALB/c mice were immunized intranasally with vehicle (PBS) and rIpaB (40 µg/mouse) on days 0, 14, and 28. Ten days after the last immunization (38 days), immunized mice were pre-treated with streptomycin and iron followed by oral infection with 5 × 10^7^ CFU bacteria. (**A**) Photos of the anal region of immunized groups at 24 h post-infection. The experiment was repeated three times, and one image out of three independent experiments (*n* = 6) is shown. (**B**) Body weight changes were monitored between infected and uninfected groups at different post-infection time points. Data represent mean ± SEM values from different mice samples (*n* = 5). Statistical analyses were performed with two-tailed Student’s *t*-test. ****P < 0.0001. (**C**) Mice were sacrificed at the indicated time points, and the colon lengths of the respective groups were observed. (i) Representative photograph of the colon lengths of different groups of mice. The experiment was repeated three times, and one image out of three independent experiments is shown. (ii) Bar representing the comparison of the colon length between the two groups of mice. Data represent mean ± SEM values from different mice samples (*n* = 3). Statistical analyses were performed with two-tailed Student’s *t*-test (***P < 0.001, ****P < 0.0001). (**D**) Colony-forming units (CFU) in homogenates of colon, cecum, and feces. Data represent mean ± SEM of the values from multiple animals (*n* = 3). Statistical analyses were performed by one-way ANOVA and Tukey’s post-test for multiple comparisons. ***P < 0.001, ****P < 0.0001

**Fig 8:**
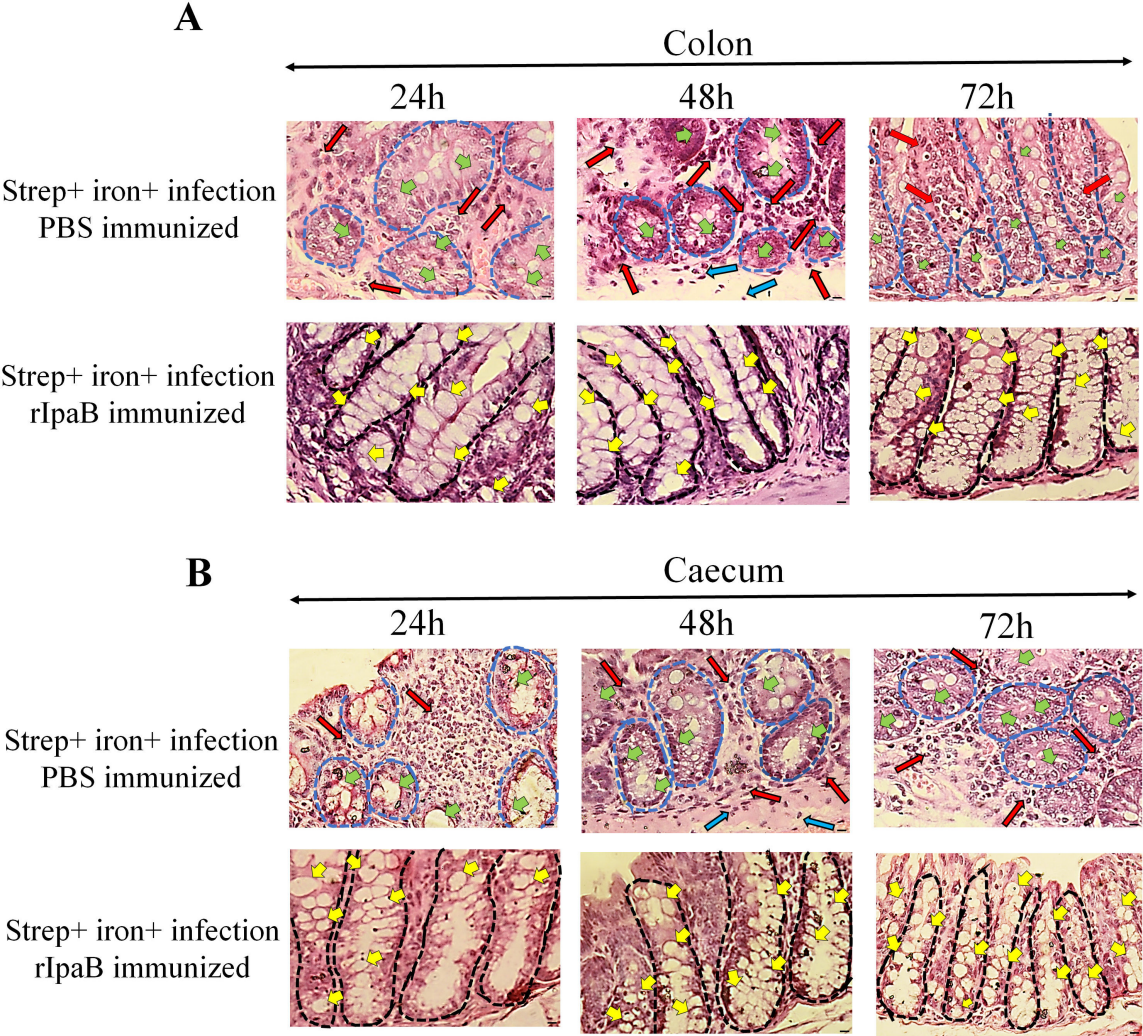
Histology sections of colon and caecum of immunized and unimmunized BALB/c mice after infection. BALB/c mice (*n* = 10) were immunized intranasally with Vehicle (PBS) and rIpaB (40µg/mouse) on days 0, 14, and 28. Ten days after the last immunization (38d), immunized mice were pre-treated with streptomycin and iron followed by oral infection with 5 × 10^7^ CFU bacteria. Mice were sacrificed at the indicated time points. Colon (**A**) and caecum (**B**) were excised, fixed, and embedded in paraffin. Tissue sections were stained with hematoxylin & eosin and observed in a microscope (40x magnification, scale bar = 10 µm). The images are the magnified form of the 10x images (areas of green box) presented in the supplemental material. Intact crypt architecture with abundant records of goblet cells, intact mucosa and submucosa without abnormal infiltrates were observed in the rIpaB immunized group of mice. PBS immunized group showed loss of crypt architecture with decreased goblet cells and increased infiltration of lymphocytes in the mucosa and submucosa. Black round dotted circles represent intact crypt architecture and blue round dotted circles represent loss of crypt architecture. Different colors of arrow indicates different parameters as follows: yellow arrow, abundant goblet cells; green arrow, loss of goblet cells; red arrow, lymphocyte infiltration in the mucosa; blue arrow, lymphocyte infiltration in the submucosa.

To investigate if rIpaB could reduce the disease severity of *Shigella flexeneri 2a* infection, immunized mice were challenged orally with a sublethal dose (5 × 10^7^ CFU) of the bacterium that was used for the model development. Vehicle-immunized mice were visibly sick with diarrhea at 24 h post-infection, while the rIpaB vaccine-immunized groups were agile and symptom-free (Fig. 7A). Mice immunized with rIpaB showed increased body weight as opposed to reduced body weight of the vehicle-immunized, infected group (Fig. 7B). Significant shortening of the colon was observed in the latter group, while rIpaB-immunized mice displayed normal colon lengths (Fig. 7C, panels i and ii). The above manifestations corroborated with *S. flexeneri 2a* colonization of the cecum and colon and shedding in the feces with significant reduction of the CFU counts in rIpaB-vaccinated mice (Fig. 7D). Together the above results suggested that the IpaB vaccine candidate might have induced immune response in the large intestine to prevent *Shigella* infection. To further investigate if this led to reduced tissue destruction in rIpaB-vaccinated mice, histopathology of the large intestinal tissues was performed. were observed in. While mice of the unimmunized (PBS treated) group showed destruction of the epithelial lining, loss of crypt architecture with decreased number of goblet cells, increased infiltration of lymphocytes in the mucosa and submucosa, and submucosal edema, the immunized (rIpaB treated) mice had intact epithelial lining and crypt architecture with abundant goblet cells, absence of mucosal or submucosal damage and no abnormal cellular infiltrates (Fig. 8A and B; Fig. S10). Overall, these results suggested that intranasal immunization with rIpaB prevented *Shigella* from colonizing the large intestine after oral infection, thereby reducing tissue damage and disease manifestations.

### Intranasal immunization with recombinant chimeric protein, IpaB–T2544, protects mice against oral *Shigella* and typhoidal *Salmonella* infections

We had previously reported that a candidate vaccine formulation based on T2544, administered subcutaneously, induced high levels of serum antibodies and robust effector and memory T-cell response, leading to protection from *S*. Typhi challenge (30). Moreover, T2544 used as a carrier protein acted as a vaccine adjuvant and augmented the immunogenicity of *S*. Typhimurium O-specific polysaccharide (39). Hence, to develop a bivalent vaccine formulation against *Shigella* and *Salmonella* spp., a chimeric protein containing T2544 and IpaB was generated. To this end, *Shigella flexneri 2a ipab* and *Salmonella* Typhi *t2544* genes were cloned in-frame, which was confirmed by agarose gel electrophoresis of the restriction digestion products (Fig. S9A), followed by nucleotide sequencing (Fig. S9B). The recombinant protein (rIpaB–T2544, 55 kDa) was purified from *E. coli* BL21 (DE3) PlyS by affinity chromatography using Ni-NTA agarose (Fig. S9C), and the purity and size confirmation was obtained by Western blots using His-tag antibody (Fig. S9D). The secondary structure of rIpaB–T2544 was analyzed by Far-UV CD spectra that showed one negative band, indicating alpha helix, and one positive band, suggesting β helical structure at 222.5 and 206.0 nm, respectively (Fig. S9E).

**Fig 9:**
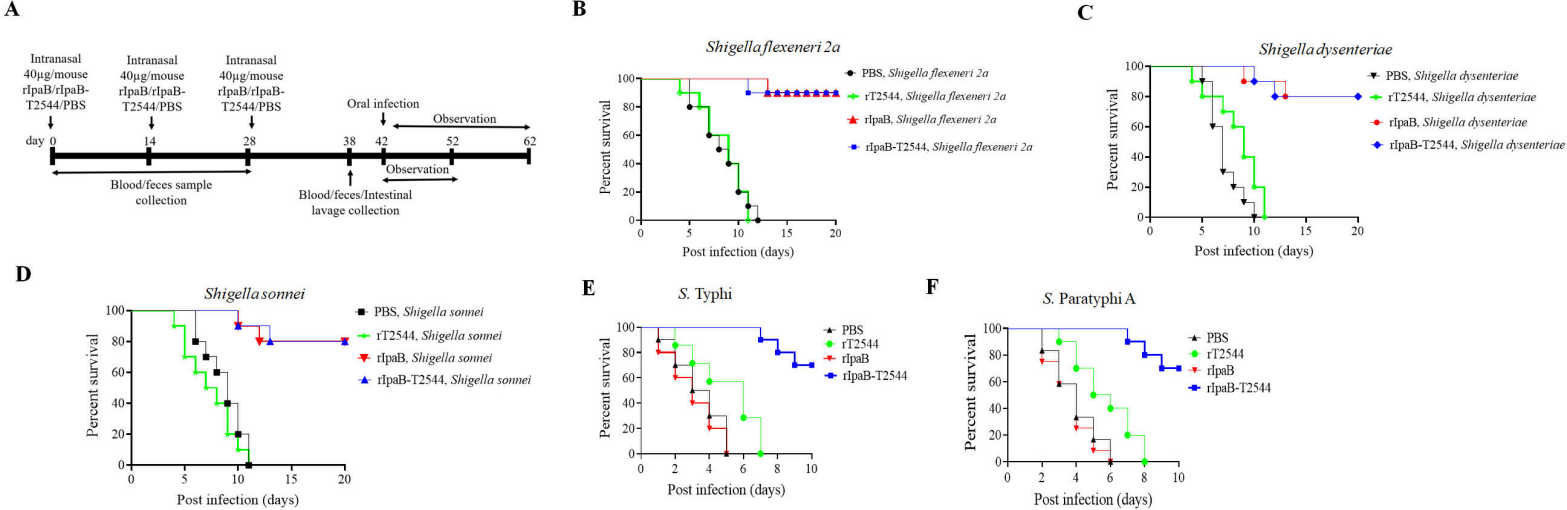
Intranasal immunization with recombinant chimeric protein, IpaB–T2544, protects mice against oral *Shigella* and typhoidal *Salmonella* infections. (**A**) Experimental schedule of immunization, sample collection, and bacterial challenge of BALB/c mice. (**B–F**) Kaplan–Meyer plot of cumulative mortality of the mice immunized intranasally with vehicle, chimeric (rIpaB–T2544), or the recombinant proteins (rIpaB and rT2544). One set of immunized mice was pre-treated with streptomycin and iron followed by oral infection with *Shigella flexeneri 2a* (5 × 10^9^ CFU, *n* = 10) (**B**), *Shigella dysenteriae* (5 × 10^8^ CFU, *n* = 10) (**C**), and *Shigella sonnei* (5 × 10^8^ CFU, *n* = 10) (**D**) and monitored for 20 days. Other sets of immunized mice were pre-treated with iron followed by oral infection with *S.* Typhi (5 × 10^7^ CFU, *n* = 10) (**E**) and S. Paratyphi A (5 × 10^5^ CFU, *n* = 10) (**F**) and monitored for 10 days. The color scheme used to mark different experimental groups are as follows: black, PBS; green, rT2544; red, rIpaB; blue, rIpaB–T2544.

To check for the protective efficacy of the candidate vaccine against *Shigella* and *Salmonella*, BALB/c mice were immunized intranasally with three doses of the recombinant chimeric protein or the individual proteins that constituted the chimera or the vehicle (PBS) at 14-day intervals (Table S4; Fig. 9A). A 10× LD_50_ oral infection dose of *Shigella* spp. killed all the mice that received either the vehicle or rT2544 within 14 days of infection, whereas 80%–90% of the mice immunized with rIpaB–T2544 or rIpaB alone were still alive beyond 20 days (Fig. 9B through D). Vaccine efficacy against *S*. Typhi and *S*. Paratyphi A, as evaluated in the BALB/c mouse model, showed nearly 70% protection following rIpaB–T2544 immunization against 10× LD_50_ doses of *S*. Typhi (5 × 10^7^ CFU) or *S*. Paratyphi (5 × 10^5^ CFU), while T2544 alone was non-protective with all the immunized mice being dead within 5–6 days (Fig. 9E and F). This suggested that IpaB acted as an immune adjuvant to T2544 for intranasal vaccination. Moreover, rIpaB–T2544 is a bivalent vaccine candidate against *Shigella* spp. and *Salmonella* (*S*. Typhi and *S*. Paratyphi A), when administered through the intranasal route.

### IpaB in the chimeric vaccine candidate augmented serum and mucosal antibody response to T2544, while rIpaB–T2544 induced a balanced T helper-cell response

**Fig 10:**
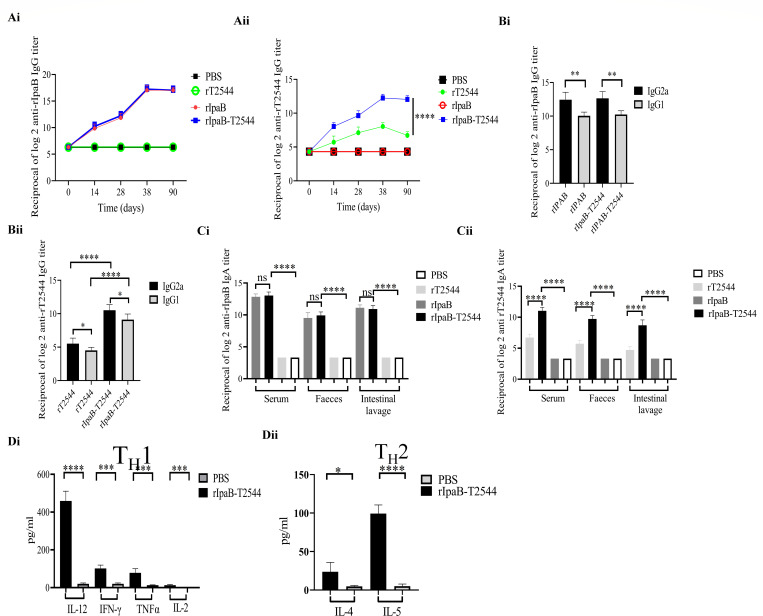
Humoral and mucosal adjuvanticity of rIpaB and balanced TH1 and TH2 cytokine production from splenocytes of intranasal rIpaB–T2544-immunized mice. BALB/c mice were immunized intranasally with vehicle (PBS) and rIpaB–T2544 (40 µg/mouse) on days 0, 14, and 28. (**A**) Time kinetics of antigen-specific total IgG in the mouse serum as measured by ELISA. Data represent mean ± SEM values from different mice samples (*n* = 5). *X*-axis indicate the time points after the start of the immunization when samples were collected. Statistical significance between the titer values from rIpaB–T2544 and rT2544 vaccine recipients is shown. Statistical analysis was performed using two-way ANOVA and Tukey’s post-test for multiple comparisons. ****P < 0.0001. The color scheme used to mark different experimental groups are as follows: black, PBS; green, rT2544; red, rIpaB; blue, rIpaB–T2544. (**B**) Serum IgG isotypes measured by ELISA at day 38. Data represent mean ± SEM values from different mice samples (*n* = 5). Statistical analysis was performed using two-tailed Student’s *t*-test. ****P < 0.0001. (**C**) ELISA showing serum IgA and intestinal sIgA titers after immunization with different antigens. Mice were sacrificed on day 38 and samples were collected. Data represent mean ± SEM values from different mice samples (*n* = 5). Statistical analysis was performed using two tailed Student’s *t*-test. ****P < 0.0001. (**D**) Immunized mice were sacrificed on day 38, and splenocytes were isolated and cultured in the presence of antigen stimulation for 48 h. (i and ii) T-cell cytokines were measured from the culture supernatants by ELISA. Data represent mean ± SEM values from different mice samples (*n* = 4). The experiment was replicated three times, and data from a representative experiment are shown. Statistical analysis was performed using two tailed Student’s *t*-test. *P < 0.05, **P < 0.01, ***P < 0.001, ****P < 0.0001.

Analysis of serum antibody endpoint titers showed that immunization with rIpaB and rIpaB–T2544 induced similar magnitudes of IpaB-specific IgG, while T2544-specific IgG titers were significantly higher for the chimeric vaccine (Fig. 10A, panels i and ii). The IgG isotypes were composed of IgG1 and IgG2a, indicating induction of both type I and type II responses, although the latter response was predominant (Fig. 10B, panels i and ii). Serum and intestinal secretory IgA titers followed the same pattern as serum IgG, with identical anti-IpaB titers for rIpaB and rIpaB–T2544 immunizations, but significantly higher anti-T2544 IgA titers for the chimeric protein vaccine (Fig. 10C, panels i and ii). Collectively, these results suggested that rIpaB–T2544 was strongly immunogenic, and IpaB acted as an immune adjuvant to T2544, significantly boosting the antibody titers in the serum and intestinal secretions.

Cell-mediated immune responses may significantly contribute to vaccine-induced protection, with Th1 response being critical for clear intracellular pathogens and Th2 cells promoting serum antibodies and sIgA production. To assess the cellular immune responses induced by our candidate vaccine, splenocytes isolated from the immunized mice were subjected to mixed leukocyte reaction and cultured for 48 h in the presence of antigen stimulation. Both Th1 (IL12, IFN-γ, TNF-α, and IL-2) and Th2 (IL-4 and IL-5) cytokines, as measured in the culture supernatants, were significantly elevated in rIpaB–T2544-immunized mice, as opposed to minimal rise in the vehicle-immunized group (Fig. 10D, panels i and ii). These results suggested a balanced T-cell response after immunization with rIpaB–T2544.

## DISCUSSION

In this study, we report, to the best of our knowledge, the first murine model of oral *Shigella* infection in a wild-type mouse strain. Iron overload BALB/c mice pre-treated with streptomycin were susceptible to *S. flexneri 2a* infection of the large intestine, leading to severe diarrhea and loss of body weight, colonic inflammation with shortening of the length, neutrophil infiltration, loss of goblet cells, crypt and villus erosions, and enhanced secretion of inflammatory cytokines and chemokines. This model was also useful for the efficacy study of a candidate vaccine against *Shigella*.

Development of an animal model of *Shigella* infection has always been challenging due to the human-restricted nature of the pathogen. Since mice are naturally resistant to *Shigella* infection, earlier attempts were directed toward other small animals. Guinea pig keratoconjunctivitis was the first reported model (9), which was useful to differentiate a virulent from a non-virulent strain of *Shigella* and indicated the role of T3SS as the virulence determinant of *Shigella* infection. However, it threw little light on host–pathogen interactions because of the irrelevant target tissue. Subsequent models that were developed, such as the rabbit ileal loop (8) and rabbit (13) or guinea pig rectal (14) infection model, offered more insights into human shigellosis. Especially, the infant rabbit model closely recapitulated the human, commonly observed with *S. dysenteriae* type I infection (rarely found nowadays) and manifested by severe inflammation, massive ulceration of the colonic mucosa, bloody diarrhea, and marked weight loss, although the authors used more frequently isolated *S. flexneri 2a* for infection (13). Notwithstanding, the above models remained non-physiological due to a different portal of entry for the bacteria. Despite having significant potential to help new drug development, they have limited usefulness for the study of protective host immune responses and development of new vaccines. Attempts were also made by manipulating the mouse intestinal environment or genetic background to establish a *Shigella* infection model. Elimination of the gut microbiota of C57BL/6 mice using an antibiotic cocktail before and after the oral challenge with *Shigella* helped to establish the infection and colitis (41), but the bacteria remained non-invasive, precluding host–pathogen interaction studies. While pre-treatment of mice with a single oral dose of streptomycin was successful to model *S*. Typhimurium colitis (17), similar attempts gave rise to predominantly small intestinal infection with few epithelial lesions and minimal PMN infiltration for oral *Shigella* infection (42), especially with *S. sonnei*. However, susceptibility to shigellosis (*S. flexneri*) dramatically improved for mice genetically deficient in NAIP-NLRC4 inflammasome, giving rise to disease manifestations that follow human infection, and the model was used to demonstrate host-protective function for inflammasomes in intestinal epithelial cells (16).

When *Shigella* invades the host cell, it must rapidly adjust to a new environment where it has to derive certain nutrients or metabolic cofactors, such as oxygen, carbon sources, and iron, which are under the host’s control. Because free iron is intrinsically toxic, the host has evolved a variety of defense mechanisms, such as the expression of iron-binding proteins to reduce the amount of iron present inside the cells. However, *Shigella* expresses molecules that can take up intracellular iron, including siderophores, heme transporters, and ferric and ferrous iron transport systems (43). *Shigella* spp. use iron for several essential functions, including respiration and DNA replication (19). The role of iron in *Shigella* pathogenesis is underscored by marked upregulation of the iron acquisition systems (Iut, Sit, FhuA, and Feo) and the stress-associated Suf protein in the face of iron starvation by intracellular bacteria (44). In environments where oxygen is scarce, like the gut lumen, feO supplies iron. The Sit system is specifically designed to supply iron to the bacteria growing inside the host cells, since it is expressed in the presence of oxygen and carries ferrous iron (25). The outer membrane receptor needed for aerobactin (siderophore of *Shigella*) import is encoded by the iutA gene. Iron-loaded aerobactin can enter the cell using the general hydroxamate transport system, FhuBCD, after passing through the outer membrane (45). We established a mouse model of *S. flexneri* in the wild-type background by combining streptomycin pre-treatment with iron overload.

In our study, the pathogenic lesions in the large intestine of the infected mice recovered by day 7, which corresponded to the average disease duration in humans (46). However, other animal models reported faster recovery with more rapid elimination of the bacteria. Rectal biopsy of patients revealed two types of colitis after *Shigella* infection, mild and moderate/severe (47). Histopathological examination of mild colitis showed a flattened surface epithelium with erosions, increased cellular infiltration in the lamina propia, absent crypt abscesses, and mild mucosal and submucosal edema. In contrast, moderate colitis was characterized by mucosal damage with crypt abscesses and dense neutrophil infiltration into the lamina propria. Histological features in our study were more similar to mild colitis in the human samples. An additional strength of our model was that it worked for multiple pathogenic *Shigella* species. Other mouse models reported are less useful because of the absence of intestinal disease in some cases (48) and the requirement of technical expertise in others (49).

We established productive infection of *S. flexneri* in BALB/c mice. Previous studies reported that intestinal epithelial-specific NAIP-NLRC4 inflammaosome activation protects C57BL/6 mice against *Shigella* infection (16). However, C57BL/6 is a Th1-biased strain, while NLRP3 inflammasome inhibition protects BALB/c mice, which is a Th2-biased strain from human metapneumovirus (50) and *Leishmania* infection (51, 52). *S. flexneri* type III secretion system effector protein IpaH7.8 E3 ubiquitin ligase plays a pivotal role in NLRP3 inflammasome activation in macrophages (53). Studies had shown that Th2 response was associated with the production of IL-4, IL-5, and IL-13 cytokines that increased disease susceptibility and persistence in mice (54, 55).

Our model is a useful adjunct to the existing models since it is simple, cheap, uses physiological route of infection and appropriate for vaccine efficacy studies . Absence of a suitable animal model has significantly hindered the development of *Shigella* vaccine that is urgently required for the developing world. We report here the construction of a subunit vaccine based on the invasin protein IpaB that is conserved across the major pathogenic *Shigella* spp. (*S. flexeneri 2a*, *S. dysenteriae*, and *S. sonnei*) and was incorporated in several vaccine preparations (35, 56). Furthermore, we developed a chimera of IpaB and a conserved outer membrane protein, T2544, from *Salmonella* Typhi and *Salmonella* Paratyphi A (30). We had previously reported that s.c. immunization with recombinant T2544 and a glycoconjugate of T2544 and *S*. Typhimurium OSP conferred protection against multiple *Salmonella* serotypes (39). Serum anti-IpaB endpoint IgG titer (1/204,800) found in this study was comparable, while serum and fecal IgA titers (1/12,800 and 1/1,280) were significantly higher compared with the previous reports. Intranasal immunization with a mixture of IpaB and IpaD resulted in IpaB-specific serum IgG and fecal IgA endpoint titers of 1/1,000,000 and 1/1,000, respectively, after three doses (35). IpaB-specific serum IgG and fecal IgA endpoint titers were found to be 1/1,000,000 and 1/100 following immunization with chimeric ipaB-IpaD along with double mutant LT (dmLT) (56). Another study found that when adjuvant rGroEL was added to rIpaB, the resulting IgG and IgA antibody titers increased by 1.5 and 1.3 times, respectively, in comparison to rIpaB alone (57). The mixture of IpaB/IpaD showed 90% and 30% protection against a 11- and 24-median LD_50_ dose of *Shigella flexeneri 2a*, while 80% and 50% protection was observed against a 5- and 9-median LD_50_ dose of *Shigella sonnei* (35). Mice immunized with IpaB-IpaD fusion protein containing dmLT confers 70%, 100%, and 40% protection against lethal doses of *S. flexeneri*, *S. sonnei*, and *S. dysenteriae* (56). On the other hand, rIpaB + GroEL-immunized mice showed 80% protection against the lethal doses of *S. flexeneri*, *S. sonnei*, and *S. boydii* (57). In our study, the vaccine chimera, rIpaB–T2544, was equally efficacious, where 90% protection was found for *S. flexeneri* and 80% protection was found for *S. sonnei* and *S. dysenteriae* with 10× LD_50_ dose, which were similar to the protection observed in the pulmonary infection model (35, 57). An interesting finding for our study was the 16-fold increase in T2544-specific IgG antibody titers in the mice immunized with the rIpaB–T2544 chimera antigen compared with rT2544 administered as a sole antigen. In addition, T2544-specific IgA titer was 40-fold higher in the serum and 16-fold higher in the fecal and intestinal washes. In the protection study, rIpaB–T2544-immunized mice showed 70% protection against *Salmonella* Typhi and Paratyphi A, while no protection was found with rT2544 alone. These results suggested that rIpaB acts as an adjuvant that enhances the immunogenicity of T2544.

IFN-γ production is required for the protection of mice against *Shigella* infection (58). *Shigella* replicates intracellularly and multiplies further when IFN-γ is not present, indicating that an IFN-γ-mediated mechanism is in place to limit infection (59). Production of IFN-γ in response to IpaB was demonstrated using peripheral blood mononuclear cells from infected individuals (34) or other vaccine candidates (35, 56, 57). In our study, mice immunized with rIpaB–T2544 enhanced IFN-γ production along with other cytokines (IL-12, TNF-α, and IL-2), indicating Th1 lymphocytes activation.

A potential limitation of our mouse model is the requirement for antibiotic pre-treatment, which alters the gut microbiota. Iron further alters the resident microbial population. This indicates that the model is not suitable to study *Shigella* infection in the context of microbial communities. As *Shigella* infection globally occurs due to the predominance of the *Shigella flexneri* strain, other strains of *Shigella* were not used for the model establishment in our study. Furthermore, for the model development and the potential of its use in vaccine-induced protection studies, we used virulent *Shigella* spp. only. *Shigella* pathogenesis is attributable to a multitude of virulence factors that enable the bacteria to invade and proliferate within the colonic epithelial cells and evade host immune responses. Most known virulence factors of *Shigella* are encoded by a large (200-kbp) virulence plasmid, which is essential for *Shigella* pathogenicity (11–13). All the strains used in our study contained the virulence plasmid, as revealed by the positive Congo red stain. Many of the virulence factors of *Shigella*, such as IcsA/VirG, are essential for pathogenesis, rendering the mutants avirulent. It would be important to study if the virulence factors that serve a non-redundant role during human and some other animal infections (14, 16) are also critical for pathogenesis in the novel mouse model developed by us. This study will inform us if this mouse model could be useful for mechanistic studies into human shigellosis. However, this is out of scope of the current article. We did not examine *Shigella* pathogenesis in terms of vacuolar escape or cell-to-cell spread in the intestinal epithelium. IpaB is a known immunogenic protein and has been studied as a candidate vaccine for tests in a lethal pulmonary mouse model; however, we did not repeat the protective efficacy study for IpaB in the mouse lung model. Furthermore, we did not compare the immune cells population activated by IpaB or IpaB–T2544. Instead, we only determined the vaccine efficacy of IpaB and IpaB–T2544 against *Shigella* spp. In our preclinical study, we have used the intranasal route of immunization, since intranasal delivery of IpaB and IpaD were reported to confer better protection in comparison with the intradermal delivery in mice (60). Although intranasal immunization with native invaplex was well tolerated and immunogenic in phase 1 studies (61, 62), one potential limitation was the failure of protection in a CHIM trial (63).

## Data Availability

The raw data supporting the conclusions of this article will be made available by the authors without undue reservation.

## AFTER EPUB

*[This article was published on 18 December 2024 with errors in the Fig. 8 caption and funding information. The caption and funding information were corrected in the current version, posted on 31 January 2025.]*

## SUPPLEMENTAL MATERIAL

Supplemental figures iai.00346-24-s0001.tif Fig. S1 and S2.

Fig. S3 iai.00346-24-s0002.tif Histology sections of the colon and caecum tissue of uninfected and untreated BALB/c mice.

Fig. S4 iai.00346-24-s0003.tiff Histology sections of the colon tissue of BALB/c mice after different treatments.

Fig. S5 iai.00346-24-s0004.tiff Histology sections of the caecum tissue of BALB/c mice after different treatments.

Fig. S6 iai.00346-24-s0005.tiff Histology sections of the colon tissue of BALB/c mice after infection.

Fig. S7 iai.00346-24-s0006.tiff Histology sections of the caecum tissue of BALB/c mice after infection.

Fig. S8 iai.00346-24-s0007.tif Histological scores of the colon and caecum tissue from BALB/c mice after infection.

Fig. S9 iai.00346-24-s0008.tif Cloning and purification of rIpaB-T2544.

Fig. S10 iai.00346-24-s0009.tiff Histology sections of colon and caecum of immunized and unimmunized BALB/c mice after infection.

Supplemental tables iai.00346-24-s0010.docx Tables S1 to S4; Supplementary figure legends and supplementary references.

ASM does not own the copyrights to Supplemental Material that may be linked to, or accessed through, an article. The authors have granted ASM a non-exclusive, world-wide license to publish the Supplemental Material files. Please contact the corresponding author directly for reuse.
