# Supplementary figures and images for: A newly developed oral infection mouse model of shigellosis for immunogenicity and protective efficacy studies of a candidate vaccine
Source: Infect Immun. 2024 Dec 18;93(1):e00346-24. doi: 10.1128/iai.00346-24 (PMC11784180; doi:10.1128/iai.00346-24)

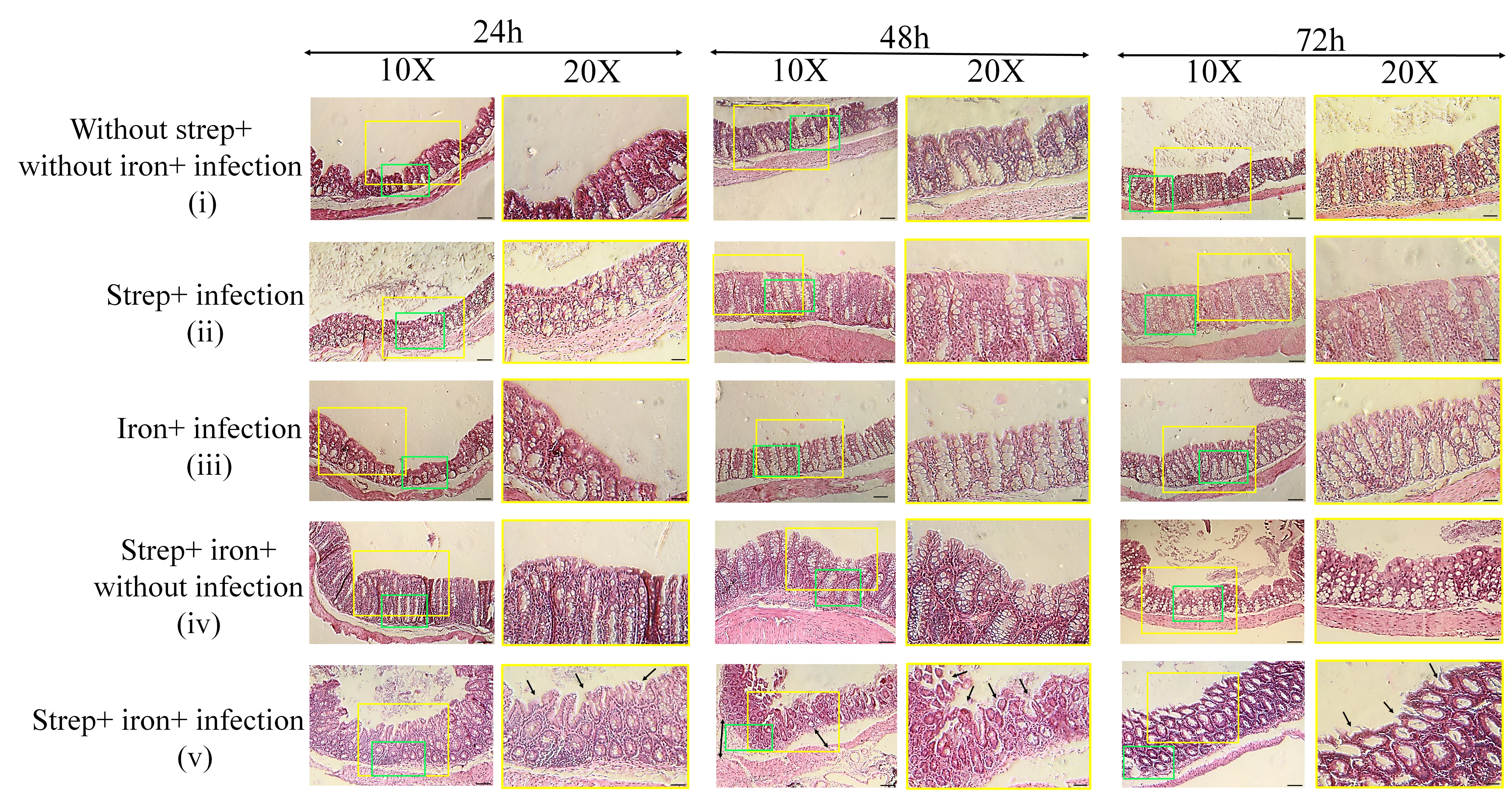

Supplement: Fig. S5 — Histology sections of the caecum tissue of BALB/c mice after different treatments. [file iai.00346-24-s0004.tiff]

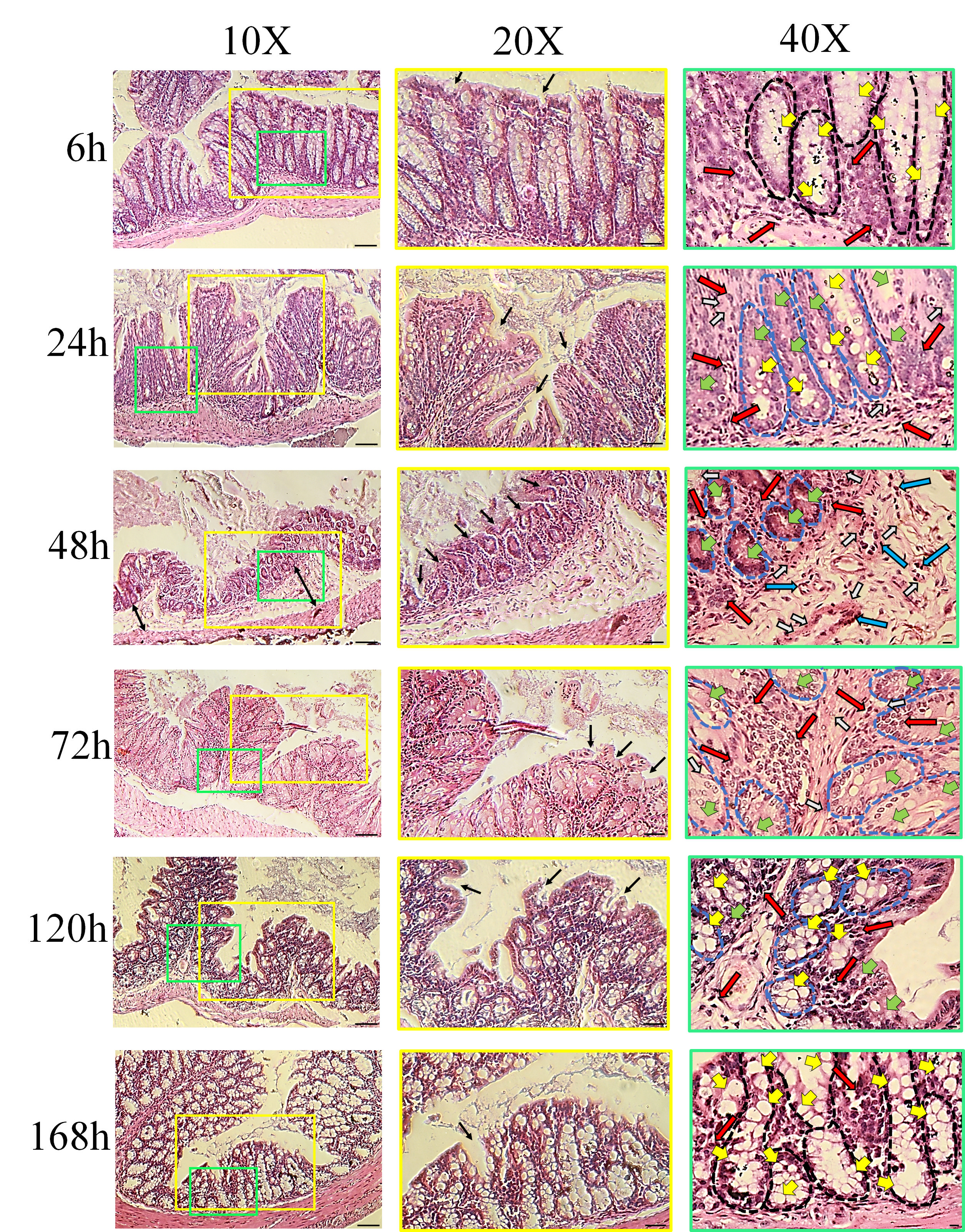

Supplement: Fig. S6 — Histology sections of the colon tissue of BALB/c mice after infection. [file iai.00346-24-s0005.tiff]

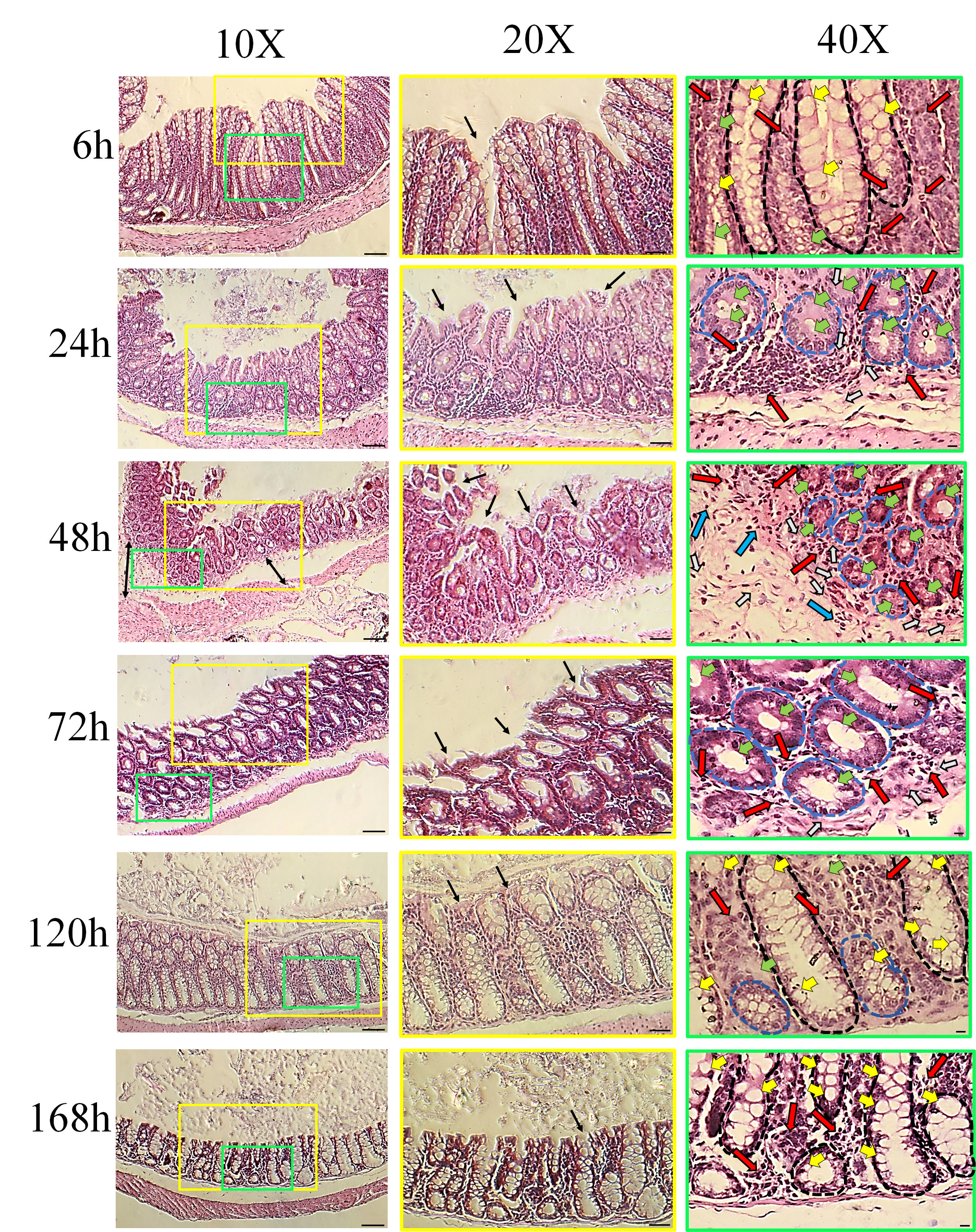

Supplement: Fig. S7 — Histology sections of the caecum tissue of BALB/c mice after infection. [file iai.00346-24-s0006.tiff]

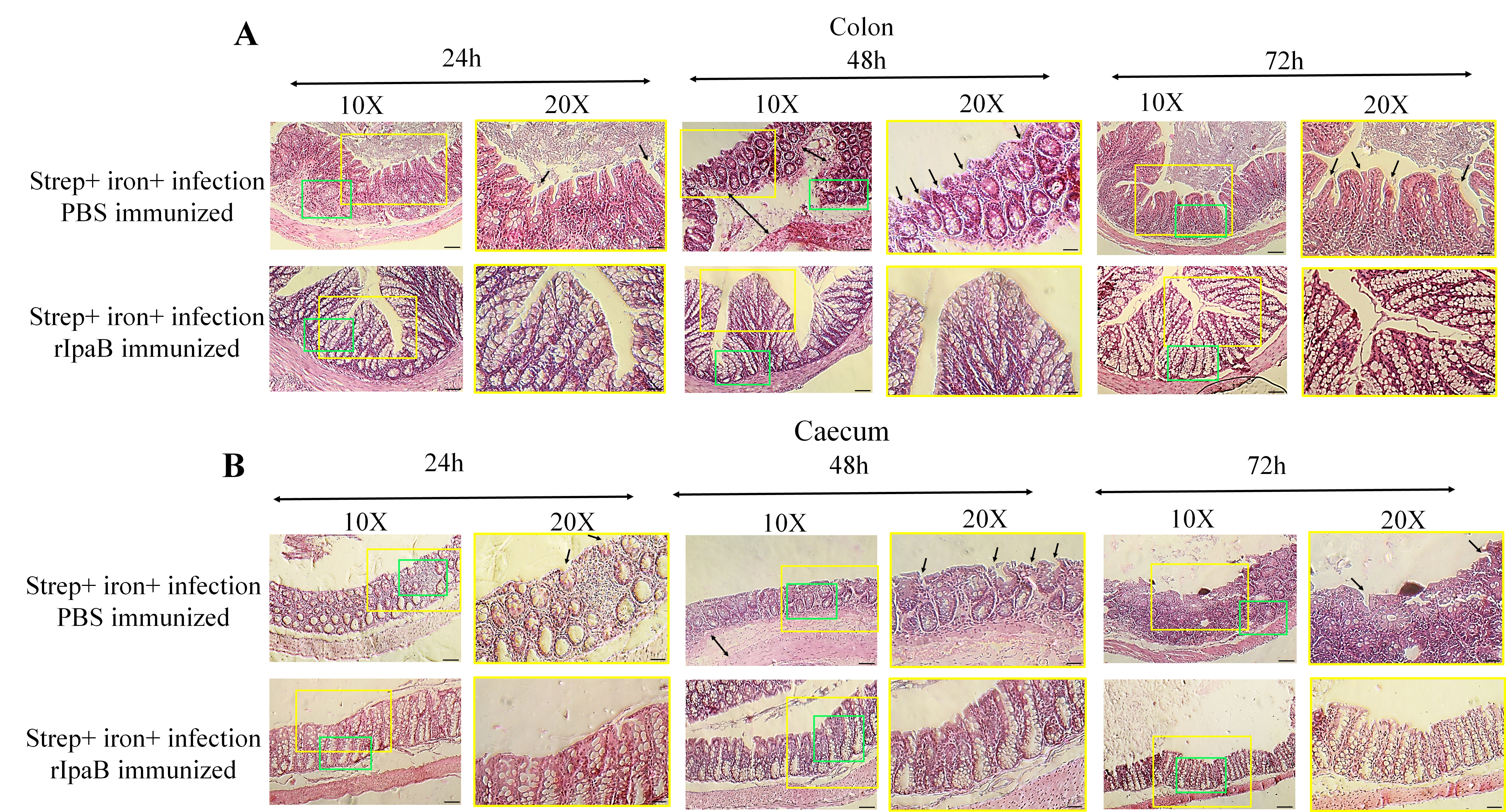

Supplement: Fig. S10 — Histology sections of colon and caecum of immunized and unimmunized BALB/c mice after infection. [file iai.00346-24-s0009.tiff]
