# Supplementary material for: A newly developed oral infection mouse model of shigellosis for immunogenicity and protective efficacy studies of a candidate vaccine
Source: Infect Immun. 2024 Dec 18;93(1):e00346-24. doi: 10.1128/iai.00346-24 (PMC11784180; doi:10.1128/iai.00346-24)
Supplement: Supplemental tables — Tables S1 to S4; Supplementary figure legends and supplementary references. [file iai.00346-24-s0010.docx]

**Supplementary Materials**

**Supplementary Figure legends**

**Supplementary Figure 1. Oral infection with 5×10^8^ cfu doses of *S. flexneri 2a* increases body weight loss, and stool shedding.** BALB/c mice were orally infected with 5×10^7^ cfu and 5×10^8^ cfu doses of *S. flexneri 2a.* (**A**) Body weight changes post-infection. Data represent mean ± SEM values of multiple animals (n = 12 for the lower dose group; n ≥ 6 for the higher dose group) at each time point. Statistical analyses were performed by two-way ANOVA, ****P < 0.0001. (**B**) Colony forming units (CFU) of *S. flexneri 2a* in the feces of infected mice. Fecal homogenates were cultured overnight on TSA plates. Data represent mean ± SEM of the values from multiple animals (n=6). Statistical analyses were performed by two-way ANOVA, *P < 0.05, **P < 0.01, ***P <0.001, ****P <0.0001. Statistical analyses were performed by student t-test (*P < 0.05, **P < 0.01, ***P <0.001).

**Supplementary Figure 2. Survival assay.** Kaplan-Meyer plot of cumulative mortality of infected mice. Streptomycin and iron pre-treated BALB/c mice (n=10) were infected with different doses of *Shigella* serovars and observed for 20 days. The colour scheme used to mark different experimental groups are as follows: uninfected-black; *Shigella flexeneri 2a*-green; *Shigella dysenteriae*-red; *Shigella* *sonnei-*blue.

**Supplementary Figure 3. Histology sections of the colon and caecum tissue of uninfected and untreated BALB/c mice.** Un treated (without strep+ without iron) + uninfected (without infection) mice (n=4) were sacrificed at 0h. Colon and caecum were excised, fixed, and embedded in paraffin. Tissue sections were stained with Hematoxylin & Eosin and observed in a microscope. Intact epithelial lining, intact crypt architecture with abundant records of goblet cells, intact mucosa and submucosa without abnormal infiltrates were observed in the group of mice. Yellow boxes represent the 20X (Scale bar =50 µm) and green boxes represent 40X (Scale bar =10 µm) magnification of the 10X (Scale bar =100 µm) image. Black round dotted circles represent intact crypt architecture, yellow arrow represents- abundant goblet cells.

**Supplementary Figure 4. Histology sections of the colon tissue of BALB/c mice after different treatments.** Different groups of mice (n=6 mice per group) were infected with 5×10^7^ CFU *Shigella flexeneri 2a* and sacrificed at the indicated time points. Colon was excised, fixed, and embedded in paraffin. Tissue sections were stained with Hematoxylin & Eosin and observed in a microscope. Intact epithelial lining was observed in the control group of mice (i-iv, 20X magnification). Streptomycin-iron with infected group (v) showed degenerative changes in the epithelial lining (20X magnification), and submucosal swelling (edema) (10X magnification). Yellow boxes represent the 20X (Scale bar =50 µm) magnification of the 10X (Scale bar =100 µm) image. Green boxes indicate the region selected for presentation in Figure 4 (colon). Double-headed black arrow indicates submucosal swelling (edema); single black arrow indicates the changes in the epithelial lining.

**Supplementary Figure 5. Histology sections of the caecum tissue of BALB/c mice after different treatments.** Different groups of mice (n=6 mice per group) were infected with 5×10^7^ CFU *Shigella flexeneri 2a* and sacrificed at the indicated time points. Caecum was excised, fixed, and embedded in paraffin. Tissue sections were stained with Hematoxylin & Eosin and observed in a microscope. Intact epithelial lining was observed in the control group of mice (i-iv, 20X magnification). Streptomycin-iron with infected group (v) showed degenerative changes in the epithelial lining (20X magnification), and submucosal swelling (edema) (10X magnification). Yellow boxes represent the 20X (Scale bar =50 µm) magnification of the 10X (Scale bar =100 µm) image. Green boxes indicate the region selected for presentation in Figure 4 (caecum). Double-headed black arrow indicates submucosal swelling (edema); single black arrow indicates the changes in the epithelial lining.

**Supplementary Figure 6. Histology sections of the colon tissue of BALB/c mice after infection.** Mice (n=12) were pretreated with streptomycin and iron and infected with 5×10^7^ CFU *Shigella flexeneri 2a.* Infected mice were sacrificed at the indicated time points. Colon was excised, fixed, and embedded in paraffin. (A) Tissue sections were stained with Hematoxylin & Eosin, observed in a microscope and (B) histological scores were blindly assessed. Streptomycin-iron with infected group showed degenerative changes in the epithelial lining (20X magnification), loss of crypt architecture with decreased goblet cells (40X magnification), increased infiltration of lymphocytes in the mucosa and submucosa (20X magnification), and submucosal swelling (edema) (10X magnification). Yellow boxes represent the 20X (Scale bar =50 µm) and green boxes represent 40X (Scale bar =10 µm) magnification of the 10X (Scale bar =100 µm) image. Black round dotted circles represent intact crypt architecture and blue round dotted circles represent loss of crypt architecture. Different colors of arrow indicate different parameters as follows; yellow arrow- abundant goblet cells; green arrow- loss of goblet cells; red arrow- lymphocyte infiltration in the mucosa; blue arrow- lymphocyte infiltration in the submucosa; double headed black arrow- submucosal swelling (edema); single black arrow indicates the changes in the epithelial lining; white arrow-bacteria.

**Supplementary Figure 7. Histology sections of the caecum tissue of BALB/c mice after infection.** Mice (n=12) were pretreated with streptomycin and iron and infected with 5×10^7^ CFU *Shigella flexeneri 2a.* Infected mice were sacrificed at the indicated time points. Caecum was excised, fixed, and embedded in paraffin. Tissue sections were stained with Hematoxylin & Eosin, observed in a microscope and (B) histological scores were blindly assessed. Streptomycin-iron with infected group showed degenerative changes in the epithelial lining (20X magnification), loss of crypt architecture with decreased goblet cells (40X magnification), increased infiltration of lymphocytes in the mucosa and submucosa (40X magnification), and submucosal swelling (edema) (10X magnification). Yellow boxes represent the 20X (Scale bar =50 µm) and green boxes represent 40X (Scale bar =10 µm) magnification of the 10X (Scale bar =100 µm) image. Black round dotted circles represent intact crypt architecture and blue round dotted circles represent loss of crypt architecture. Different colors of arrow indicate different parameters as follows; yellow arrow- abundant goblet cells; green arrow- loss of goblet cells; red arrow- lymphocyte infiltration in the mucosa; blue arrow- lymphocyte infiltration in the submucosa; double headed black arrow- submucosal swelling (edema); single black arrow indicates the changes in the epithelial lining; white arrow-bacteria.

**Supplementary Figure 8. Histological scores of the colon and caecum tissue from BALB/c mice after infection.** Mice (n=12) were pretreated with streptomycin and iron and infected with 5×10^7^ CFU *Shigella flexeneri 2a.* Infected mice were sacrificed at the indicated time points. (A) Colon and (B) Caecum were excised, fixed, and embedded in paraffin. Tissue sections were stained with Hematoxylin & Eosin and histological scores were blindly assessed. The experiment was repeated three times and one representative data is shown. The scoring parameters were mentioned in the supplementary table 1 and supplementary table 2.

**Supplementary Figure 9. Cloning and purification of rIpaB-T2544.** (A) 1% Agarose gel electrophoresis of pET28a-*ipab*-*t2544* clone, after restriction digestion with BamHI, and SacI, SalI and XhoI. Lane M1: 1 kb DNA ladder, M2: 100bp DNA ladder, 1: Undigested clone, 2: Restriction enzymes digested clone. (B) Sequencing of the rIpaB-T2544 clone using pET forward and reverse primers. *A,* The FASTA file format of the sequences is provided. Yellow and green colors code for the recognition sites of the restriction enzyme BamHI and linker sequence (GS linker), respectively, whereas the blue color codes for the open reading frame (ORF) of IpaB. *B,* Yellow, sky, and red colors code for the recognition sites of the restriction enzymes XhoI, SalI and SacI. The green color codes for the linker sequence (GP linker), whereas the magenta color code for the open reading frame (ORF) of t2544 and the blue color code for the open reading frame (ORF) of IpaB. (C) 12% SDS–PAGE of recombinant purified proteins (5µg of each). Molecular mass markers (kDa) are on the left. (D) Western blot probed with anti-His antibody after resolving the recombinant purified proteins (rT2544, 5 µg, rIpaB, 7 µg, rIpaB-T2544, 3 µg) in 12% SDS-PAGE. Molecular mass markers (kDa) are on the left. The experiment was repeated three times and a representative blot is shown here. (E) Far-UV circular dichroism spectra of protein samples (180 µg/ml) captured at the wavelength range of 200 to 350 nm at 25°C in PBS (pH 7.4) on the Jasco-1500 spectrophotometer. Data presented as ellipticity (CD [mdeg]) after subtracting the baseline values. Different lines are describesd as follows; upper line- rT2544, middle line-rIpaB and lower line- rIpaB-T2544. Experiment was replicated three times, and data from a representative experiment are shown.

**Supplementary Figure 10. Histology sections of colon and caecum of immunized and unimmunized BALB/c mice after infection.** BALB/c mice (n=10) were immunized intranasally with Vehicle (PBS) and rIpaB (40µg/mouse) on days 0, 14, and 28. Ten days after the last immunization (38d), immunized mice were pre-treated with streptomycin and iron followed by oral infection with 5× 10^7^ CFU bacteria. Mice were sacrificed at the indicated time points. Colon and caecum were excised, fixed, embedded in paraffin, and tissue sections were stained with Hematoxylin & Eosin. Intact epithelial lining was observed in the control group of mice (i-iv, 20X magnification). Streptomycin-iron with infected group (v) showed degenerative changes in the epithelial lining (20X magnification), and submucosal swelling (edema) (10X magnification). Yellow boxes represent the 20X (Scale bar =50 µm) magnification of the 10X (Scale bar =100 µm) image. Green boxes indicate the region selected for presentation in Figure 8. Double-headed black arrow indicates submucosal swelling (edema); single black arrow indicates the changes in the epithelial lining.

**Supplementary Tables**

**Supplementary Table 1. Histopathological scoring system for intestinal changes (1, 2, 3, 4, 5)**

| **SL NO.** | **Criteria** | **Score** |
| --- | --- | --- |
| 1 | Intact crypt architecture with abundant records of goblet cells, intact mucosa, and submucosa without abnormal infiltrates, without submucosal swelling and without degenerative changes in the epithelial lining | 0 |
| 2.1 | Loss of crypt architecture with minimal goblet cells loss (<50%) | 1 |
| 2.2 | loss of crypt architecture with mild goblet cells loss (50%) | 2 |
| 2.3 | loss of crypt architecture with moderate goblet cell loss (>50%) | 3 |
| 3.1 | PMN infiltration into mucosa without submucosa with degenerative changes in the epithelial lining | 1 |
| 3.2 | PMN infiltration into mucosa with submucosa with degenerative changes in the epithelial lining | 2 |
| 3.3 | PMN infiltration into mucosa with submucosa and submucosal swelling with degenerative changes in the epithelial lining | 3 |
|  | **Severity of parameters** | **Combined score** |
| 4.1 | No inflammation | 0 |
| 4.2 | Minimal signs of inflammation | 1-2 |
| 4.3 | Mild inflammation | 3-4 |
| 4.4 | Moderate inflammation | 5-8 |
| 4.5 | Profound inflammation | 9-13 |

**Supplementary Table 2. Histopathological scoring of colon and caecum for intestinal changes**

|  |  | Post-infection time points | | | | | |
| --- | --- | --- | --- | --- | --- | --- | --- |
| SL NO. | Criteria | 6h | 24h | 48h | 72h | 120h | 168h |
| 1 | Loss of crypt architecture with minimal goblet cells loss (<50%) |  |  |  |  | 1 |  |
| 2 | loss of crypt architecture with mild goblet cells loss (50%) |  | 2 |  | 2 |  |  |
| 3 | loss of crypt architecture with moderate goblet cell loss (>50%) |  |  | 3 |  |  |  |
| 4 | PMN infiltration into mucosa without submucosa with degenerative changes in the epithelial lining | 1 | 1 |  | 1 | 1 | 1 |
| 5 | PMN infiltration into mucosa with submucosa with degenerative changes in the epithelial lining |  |  |  |  |  |  |
| 6 | PMN infiltration into mucosa with submucosa and submucosal swelling with degenerative changes in the epithelial lining |  |  | 3 |  |  |  |
|  | Combined score | 1 | 3 | 6 | 3 | 2 | 1 |
|  | Severity of parameters | Minimal | Mild | Moderate | Mild | Minimal | Minimal |

**Supplementary Table 3. List of reagents used in this study**

| **Name** | **Company** | **Catalogue No** |
| --- | --- | --- |
| Trypticase soy agar (TSA) | BD Difco | 211043 |
| Trypticase soy Broth (TSB) | BD Difco | 211768 |
| Hectoen enteric agar (HEA) | BD Difco | 285340 |
| LB broth (Luria Bertani) | BD Difco | 244620 |
| Terrific Broth | BD Difco | 243820 |
| Ferric chloride (FeCl_3_) | LOBA Chemie | 0381700500 |
| Streptomycin sulfate | Puregene | PG-800S |
| Desferrioxamine (Desferal) | Novartis | 2210413 |
| Sodium bicarbonate (NaHCO_3_) | Sigma | S5761 |
| Gentamicin | Gibco | 15710-064 |
| Triton-X100 | Sigma | T8787 |
| FBS | Gibco | 10270-106 |
| protease inhibitor cocktail | Sigma | P8340 |
| formalin | Sigma | HT501128 |
| Hematoxylin | Himedia | S014 |
| Eosin | Himedia | S007 |
| IPTG | Sigma | 16758 |
| Urea | SRL | 62762 |
| NaCl | Amresco | X190 |
| imidazole | Omnipur | 5720 |
| Tris | Puregene | PG-7940 |
| SDS | Sigma | L4390 |
| Acrylamide-Bis-acrylamide 40% solution | Sigma | A9926 |
| Ni-NTA slurry | Qiagen | 30230 |
| Glycerol | Himedia | MB060 |
| PVDF membrane | Millipore | IPVH00010 |
| BSA | SRL | 83803 |
| Tween-20 | Himedia | MB067 |
| His-tag antibody | CST | 2365 |
| Rabbit anti-mouse IgG | Invitrogen | 31450 |
| Anti-mouse IgG1 | Sigma | R136808 |
| Anti-mouse IgG2a | Sigma | R124491 |
| goat anti-mouse IgA | Invitrogen | 62-6720 |
| TMB substrate | BD OptEIA^TM^ | 555214 |
| SuperSignal West Pico | Thermo Scientific | 34580 |
| RPMI 1640 medium | Gibco | 23400-021 |

**Supplementary Table 4. Vaccine formulation doses and bacterial strains used for the immunogenicity and challenged study**

| Mice strain | Immunogen | Route of immunization | Adjuvant | Dose  (µg/mice) | Bacterial strain for infection study | Infection dose | Infection route |
| --- | --- | --- | --- | --- | --- | --- | --- |
| BALB/c | rIpaB-T2544  rIpaB  PBS | Intranasal | Alum  Alum  Alum | 40  40  40 | *Shigella flexeneri 2a*  *Shigella dysenteriae*  *Shigella sonnei* | 5 x 10^9^ CFU  5 x 10^8^ CFU  5 x 10^8^ CFU | Oral |
| BALB/c | rIpaB-T2544  rT2544  PBS | Intranasal | Alum  Alum  Alum | 40  40  40 | *S.* Typhi | 5 x 10^7^ CFU | Oral |
| BALB/c | rIpaB-T2544  rT2544  PBS | Intranasal | Alum  Alum  Alum | 40  40  40 | *S.* Paratyphi A | 5 x 10^5^ CFU | Oral |

**References**

1. Mitchell PS, Roncaioli JL, Turcotte EA, Goers L, Chavez RA, Lee AY, Lesser CF, Rauch I, Vance RE. NAIP-NLRC4-deficient mice are susceptible to shigellosis. Elife. 2020 Oct 19;9:e59022. doi: 10.7554/eLife.59022. PMID: 33074100; PMCID: PMC7595732.
2. Barthel M, Hapfelmeier S, Quintanilla-Martínez L, Kremer M, Rohde M, Hogardt M, Pfeffer K, Rüssmann H, Hardt WD. Pretreatment of mice with streptomycin provides a Salmonella enterica serovar Typhimurium colitis model that allows analysis of both pathogen and host. Infect Immun. 2003 May;71(5):2839-58. doi: 10.1128/IAI.71.5.2839-2858.2003. PMID: 12704158; PMCID: PMC153285.
3. Erben U, Loddenkemper C, Doerfel K, Spieckermann S, Haller D, Heimesaat MM, Zeitz M, Siegmund B, Kühl AA. A guide to histomorphological evaluation of intestinal inflammation in mouse models. Int J Clin Exp Pathol. 2014 Jul 15;7(8):4557-76. PMID: 25197329; PMCID: PMC4152019.
4. Koelink PJ, Wildenberg ME, Stitt LW, Feagan BG, Koldijk M, van 't Wout AB, Atreya R, Vieth M, Brandse JF, Duijst S, Te Velde AA, D'Haens GRAM, Levesque BG, van den Brink GR. Development of Reliable, Valid and Responsive Scoring Systems for Endoscopy and Histology in Animal Models for Inflammatory Bowel Disease. J Crohns Colitis. 2018 Jun 28;12(7):794-803. doi: 10.1093/ecco-jcc/jjy035. PMID: 29608662; PMCID: PMC6022651.
5. Okada K, Itoh H, Ikemoto M. Circulating S100A8/A9 is potentially a biomarker that could reflect the severity of experimental colitis in rats. Heliyon. 2020 Feb 29;6(2):e03470. doi: 10.1016/j.heliyon.2020.e03470. PMID: 32140589; PMCID: PMC7052069.
